# Proteomic Profiling Reveals the Molecular Control of Oocyte Maturation

**DOI:** 10.1016/j.mcpro.2022.100481

**Published:** 2022-12-07

**Authors:** Hongzheng Sun, Guangyi Sun, Haotian Zhang, Huiqing An, Yueshuai Guo, Juan Ge, Longsen Han, Shuai Zhu, Shoubin Tang, Congyang Li, Chen Xu, Xuejiang Guo, Qiang Wang

**Affiliations:** 1 State Key Laboratory of Reproductive Medicine, Suzhou Municipal Hospital, Nanjing Medical University, Nanjing, China; 2 Department of Histology and Embryology, Nanjing Medical University, Nanjing, China; 3 Center for Global Health, School of Public Health, Nanjing Medical University, Nanjing, China

**Keywords:** oocyte, proteome, epigenetics, mRNA decay, ubiquitination, APC, anaphase-promoting complex, BubR1, budding uninhibited by benzimidazole-related 1, CRLs, Cullin-RING ubiquitin ligases, COC, cumulus–oocyte complexes, DEP, differentially expressed protein, FDR, false discovery rate, GV, germinal vesicle, GVBD, germinal vesicle breakdown, GO, gene ontology, HCD, higher-energy collision dissociation, LSM, RNA-binding Like-SM, MI, metaphase I, MII, metaphase II, SCF, Skp1–Cullin1–F-box complex, SAC, spindle assembly checkpoint, TMT, tandem mass tag

## Abstract

Meiotic maturation is an intricate and precisely regulated process orchestrated by various pathways and numerous proteins. However, little is known about the proteome landscape during oocytes maturation. Here, we obtained the temporal proteomic profiles of mouse oocytes during *in vivo* maturation. We successfully quantified 4694 proteins from 4500 oocytes in three key stages (germinal vesicle, germinal vesicle breakdown, and metaphase II). In particular, we discovered the novel proteomic features during oocyte maturation, such as the active Skp1–Cullin–Fbox pathway and an increase in mRNA decay–related proteins. Using functional approaches, we further identified the key factors controlling the histone acetylation state in oocytes and the vital proteins modulating meiotic cell cycle. Taken together, our data serve as a broad resource on the dynamics occurring in oocyte proteome and provide important knowledge to better understand the molecular mechanisms during germ cell development.

## Highlights

• *In vivo* quantitative and comparative analysis performed for mouse oocytes.
• CEP57L1 plays important roles for assembly of meiotic apparatus.
• SIN3A modulates histone deacetylation during oocyte maturation.
• mRNA metabolism is active in MII oocytes.
• Fbxo28 participates in substrates degradation in oocytes.

## Graphical Abstract

**Figure:**
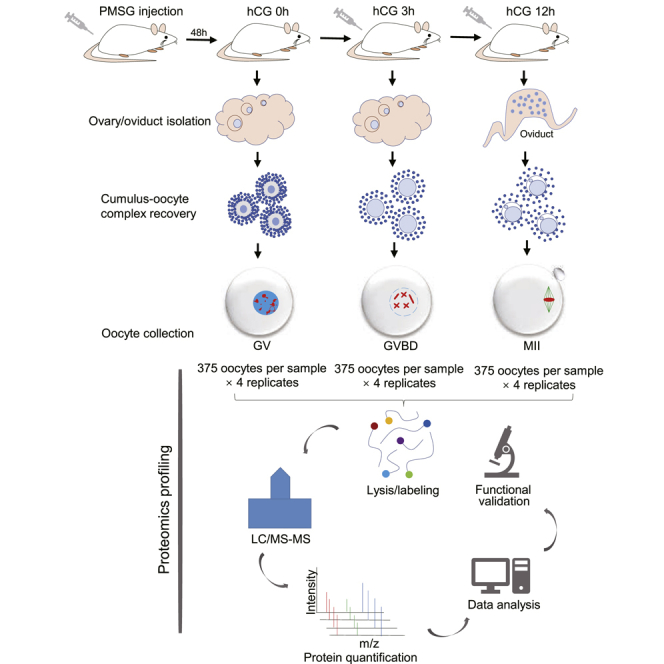

## In Brief

Using TMT-based LC-MS/MS, we systematically performed a quantitative proteomic analysis of GV, GVBD, and MII oocytes. We successfully quantified 4694 proteins from 4500 oocytes in three key stages. By combining bioinformatics analysis with functional approaches, our results showed the protein kinetics and molecular mechanisms during meiotic maturation.

In mammals, oocytes derive from embryonic precursor cells termed primordial germ cells, which migrate to the genital ridge and undergo mitosis to form primordial follicles. During fetal development, meiotic oocytes experience a lengthy arrest at the diplotene stage of meiosis I, named germinal vesicle (GV) stage. Before ovulation, oocytes undergo a long period of growth and maturation in the follicle, ranging from about 3 weeks in mice to 3 to 4 months in humans (1, 2). Immature oocytes resume meiosis I characterized by the breakdown of germinal vesicle (GVBD) upon the stimulation of gonadotropin cues. After GVBD, oocytes enter the metaphase I (MI) stage characterized by chromosome alignment on the equatorial plate and spindle organization attaching to the kinetochores (3). Then oocytes enter into anaphase I, along with the first polar body emission. Finally, the oocytes become arrested at the metaphase of the meiosis II (MII) stage awaiting fertilization.

Oocyte maturation is accompanied by numerous biological processes, such as chromosome segregation, mRNA decay, and metabolic change (4). Failure to complete any of these steps may result in meiotic defects, fertilization failure, or even embryonic arrest. Successful meiosis requires a fine regulatory network for timely degradation and translation of certain proteins to ensure the cell cycle process and chromosome integrity (5). Abnormal protein expression could lead to impaired oocyte maturation, fragmentation, and aneuploidy (6). Therefore, uncovering proteome patterns in oocytes is essential for understanding the molecular control of meiotic maturation. In previous studies, the combination of two-dimensional electrophoresis and mass spectrometry has been used to identify the proteome of MII oocytes (7, 8) the dynamic proteome during *in vivo* oocyte maturation remains unknown, mainly due to technical limitations and material scarcity.

In the present study, we performed proteome profiling of mouse oocytes at three key stages (GV, GVBD, and MII). The results showed the protein kinetics and interaction network during meiotic maturation. Furthermore, we explored the individual pathways enriched by differentially expressed proteins and conducted the functional validation of critical factors. Our findings provide a valuable resource for probing mammalian oocyte proteome, as well as molecular pathways modulating oocyte development.

## Experimental Procedures

### Mouse

All animal experiments were approved by the Animal Care and Use Committee of Nanjing Medical University and were performed according to the guidelines of the local animal ethical committee. Female C57BL/6 mice were purchased from the experimental animal center of Nanjing Medical University and were housed in ventilated cages with a standard 12 h/12 h light/dark cycle at room temperature (22 °C) under controlled humidity (20–30%).

### Oocytes Collection, Culture, and Treatment

Oocyte donor female mice (3- to 4-week-old C57BL/6) were superovulated by injecting 5 IU of pregnant mares serum gonadotropin followed by 5 IU of human chorionic gonadotropin in 48 h after pregnant mares serum gonadotropin priming. Mice were sacrificed by cervical dislocation, and oocytes at GV, GVBD, and MII stages were collected at 0, 3, or 12 h post human chorionic gonadotropin injection. To collect GV and GVBD oocytes, cumulus–oocyte complexes (COC)s were obtained by manually rupturing antral ovarian follicles, and cumulus cells were removed by repeatedly pipetting. To collect MII oocytes, COCs were harvested from the ampullae of the oviduct, and cumulus masses were removed by incubation in a hyaluronidase medium. For *in vitro* maturation, fully grown GV oocytes were cultured in an M16 medium at 37 °C in a 5% CO2 incubator under mineral oil. For subsequent analysis, oocytes at GV, GVBD, MI, and MII stages were collected, respectively, at 0, 2, 7, and 13 h. For MLN4924 treatment, fully grown GV oocytes were cultured in M16 medium with 1 μM MLN4924. For subsequent proteomic analysis, oocytes at the MI stage were harvested at 7 h.

### *In Vitro* Fertilization and Embryo Culture

*In vitro* fertilization assays were conducted as described (9). In brief, sperm was obtained from cauda epididymides of C57BL/6 male mice aged 12 weeks, followed by being capacitated for 1 h in human tubal fluid fertilization medium supplemented with 10 mg/ml bovine serum albumin. Then, dispersed sperm at a concentration of 4∗10^5^/ml were added to 100 μl human tubal fluid drops containing COCs for 6 h at 37 °C, 5% CO2. After fertilization, zygotes were cultured in 100 μl KSOM medium (Millipore, Merck) under mineral oil at 37 °C in a humidified atmosphere of 5% CO2, 5% O2, 90% N2.

### PCR Amplification and *In Vitro* Transcription

*Ubap1* open reading frame was cloned into a pCS2+ vector with six Myc tags. The forward primer contained an FseI enzyme digestion site, and the reverse primer consisted of an AscI enzyme digestion site. Capped cRNAs were synthesized from the linearized plasmid by *in vitro* transcription using SP6 mMESSAGE mMACHINE (Ambion) according to the manufacturer’s instruction.

### Immunofluorescence and Image Analysis

Hundred oocytes from three mice were collected and fixed in 4% paraformaldehyde for 30 min and permeabilized with 0.5% Triton X-100 for 15 min at room temperature (RT). After treatment with 1% bovine serum albumin–supplemented PBS for 2 h at RT, samples were incubated with the primary antibody diluted in 1% bovine serum albumin–supplemented PBS overnight at 4 ^◦^C. After washing in PBS containing 0.2% polyvinylpyrrolidone (PBS/PVP), samples were incubated with corresponding secondary antibodies for 1 h at RT. After washing in PBS/PVP 3 times (10 min per time), nuclei were stained with propidium iodide (red) or Hoechst 33342 (Hoechst, blue) for 5 min. After washing 3 times, samples were mounted on an anti-fade medium and observed by using a Laser Scanning Confocal Microscope (LSM 710, Zeiss). Multiple images were exposed to IMAGE J (NIH) for processing and intensity measurements. Intensity measurements were done on the normalized sections using the IMAGE J measure function. Data were normalized concerning background levels. Antibodies are listed in supplemental Table S6.

### Western Blotting

A total of 100 oocytes of each sample were incubated at 95 °C for 5 min in Laemmli sample buffer containing protease inhibitors and then subjected to SDS-PAGE. Proteins were electrophoresed on 10% acrylamide gels and transferred to polyvinylidene fluoride membranes (Millipore) through a semidry transfer for 1.5 h at 250 mA. Membranes were blocked in 10% nonfat milk/PBST (PBS containing 0.1% Tween 20) for 3 h at RT and then incubated with the primary antibody overnight at 4 ^◦^C. The membranes were then washed thrice for 10 min. Subsequently, the membranes were incubated with the HRP-conjugated secondary antibodies for 2 h at RT. Membranes were washed with PBST 3 times (10 min per time). Then, protein bands were visualized via Pierce ECL Pierce ECL Western Blotting Substrate (Thermo Fisher Scientific, Inc). Reagents are listed in supplemental Table S7.

### RNA Isolation and Quantitative Real-time PCR

Total RNA was extracted from oocytes (50 oocytes per sample) using the Arcturus PicoPure RNA isolation kit (Applied Biosystems) according to the manufacturer’s guidelines. First-strand cDNA synthesis was performed using a cDNA Kit (QIAGEN, Germany), followed by storage at -20 °C until use. Quantitative real-time PCR was performed using an ABI StepOne Plus real-time PCR system (Applied Biosystems) with SYBR-green PCR master mix. Experiments were performed at least in triplicate. Primer sequences are listed in supplemental Table S8.

### Microinjection

siRNAs for knockdown experiments were purchased from Gene Pharma (Shanghai, China). Individual siRNA was diluted with water to give a working concentration of 20 μM, and approximately 5 pl of the solution was injected with the Narishige microinjector. An siRNA negative control with the same volume was injected as control. After injection, GV oocytes were cultured in an M16 medium containing 2.5 M milrinone for 20 h to facilitate mRNA degradation and then cultured in a milrinone-free fresh M16 medium for further experiments. reverse transcription–quantitative polymerase chain reaction (RT-qPCR) was performed to confirm the efficiency of siRNA knockdown. siRNA sequences are listed in supplemental Table S9.

### Proteomics

Two proteomic profiles were included in this study; one was the proteomic analysis of GV, GVBD, and MII oocytes and the other was the proteomic analysis of oocytes after MLN4924 treatment and Fbxo28 knockdown. Proteomic profiles of GV, GVBD, and MII oocytes were conducted as described (4). In brief, the oocytes collected from GV, GVBD, and MII stages (4 replicates per stage, each replicate containing 375 oocytes) were lysed in urea lysis buffer (8 M urea, 75 mM NaCl, 50 mM Tris, pH 8.2, 1% mixture, 1 mM NaF, 1 mM β-glycerophosphate, 1 mM sodium orthovanadate, and 10 mM sodium pyrophosphate). The lysates were sonicated for 3 s and cooled in ice to avoid overheating. This procedure was repeated three times and then followed by centrifugation at 40,000*g* for 60 min (4 °C). The protein concentration was measured using the Bradford method. Cysteines were reduced with 5 mM DTT at 56 °C for 25 min followed by alkylation in 14 mM iodoacetamide solution for 30 min in the dark at room temperature. Unreacted iodoacetamide was quenched by DTT for 15 min. Then 25 mM Tris, pH 8.2 was added to dilute the urea. The proteins were digested overnight at 37 °C in a solution of 5 ng/μl trypsin (Promega) and terminated by trifluoroacetic acid. After purification by an OASIS HLB 1 cc Vac cartridge (Waters), the samples were subjected to 6-plex tandem mass tag (TMT) labeling in two batches for GV, GVBD, and MII oocytes or TMTpro 12-plex labeling for oocytes after MLN4924 treatment and Fbxo28 knockdown.

To improve the protein sequence coverage, the TMT-labeled peptide mixtures were separated using the high-pH reversed-phase fractionation technology based on the ACQUITY UPLC M-class system (Waters) with an BEH C18 Column (300 μm × 150 mm, 1.7 μm; Waters). A 128-min gradient (3% buffer B for 14 min, 3%–8%B for 1 min, 8%–29%B for 71 min, 29%–41% B for 12 min, 41%–100%B for 1 min, 100% buffer B for 8 min, 100%–3%B for 1 min, followed by 20 min at 3% B) was employed with buffer A (20 mM ammonium formate, pH 10) and buffer B (100% ACN). A total of 30 fractions were generated for each of TMT6 and TMTpro 12-plex experiments and then dried with a SpeedVac concentrator.

All fractions of TMT6 experiments were analyzed by liquid chromatography–tandem mass spectrometry (LC-MS/MS) using an UltiMateTM 3000 RSLCnano HPLC system coupled through a nanoelectrospray source to LTQ Orbitrap Velos mass spectrometer (Thermo Fisher Scientific). Peptides were separated with a trap column (75 μm × 2 cm, Acclaim PepMap100 C18 column, 3 μm, 100 Å; Thermo Fisher Scientific) and an analytical column (75 μm × 25 cm, Acclaim PepMap RSLC C18 column, 2 μm, 100 Å; Thermo Fisher Scientific) at 300 nl/min using a 220-min gradient (3% buffer B for 5 min, 3% to 5% buffer B for 3 min, 5% to 30% buffer B for 167 min, 30% to 45% buffer B for 15 min, 45% to 99% buffer B for 1 min, 99% buffer B for 8 min, 99% to 3% buffer B for 1 min, 3% buffer B for 20 min). The parameter settings for mass spectrometry (MS) could be found in our previously published paper (10). Briefly, the MS1 scan was obtained for an *m/z* range 400 to 1800 at a resolution of 60,000, and a low-energy MS/MS scan in the linear ion trap (collision induced dissociation) followed by a higher-energy MS/MS scan in the octopole collision cell (higher-energy collision dissociation, HCD) was acquired for the eight most intense ions from the survey scan. Dynamic mass exclusion windows of 60 s were used.

All fractions of the TMTpro 12-plex experiment were analyzed using an Orbitrap Fusion Lumos mass spectrometer (Thermo Fisher Scientific) coupled to a Proxeon Easy-nLC 1200 system. Peptides were separated on an analytical column (75 μm × 160 mm, 1.9 μm, Dr. Maisch) using a 95-min gradient (3% to 5% buffer B for 5 s, 5% to 15% buffer B for 40 min, 15% to 28% buffer B for 34 min and 50 s, 28% to 38% buffer B for 12 min, 38% to 100% buffer B for 5 s, 100% buffer B for 8 min) at 300 nl/min. The parameter settings for MS could be found in previously published paper (11). Briefly, data were acquired with an MS1 scan for an m/z range 350 to 1500 with a resolution of 60,000 followed by data-dependent HCD MS2 spectra in the Orbitrap with a resolution of 50,000, HCD collision energy of 36%. Each scan cycle was set as 1.5 s.

### Protein Identification and Quantification

MaxQuant software (V1.2.2.5) was used to search the UniProt mouse proteomic database (downloaded on June 18, 2018; 61,293 protein sequences) for comparison of GV, GVBD, and MII oocytes mass spectrometry raw files while raw proteomic data of oocytes after MLN4924 treatment and Fbxo28 knockdown were processed with MaxQuant software (V1.6.5.0) using UniProt mouse proteomic database (downloaded on October 18, 2020; 63,755 protein sequences). Default parameters were adopted for MaxQuant software (V1.2.2.5) and (V1.6.5.0) except for the TMT settings. The false discovery rate (FDR) value for peptide and protein identification was set to 1%. The peptide spectrum match FDR was set to 1% based on target-decoy search, and only proteins with at least one specific peptide were used. The precursor mass tolerance was set to 20 ppm, and product ions were searched with a mass tolerance 0.5 Da. A maximum of two missed cleavages were allowed when searches were performed using the trypsin's enzymatic specificity. Carbamoyl methylation (+57.0215 Da) on the cysteine residue was set as static modifications. Variable modifications were defined as oxidation of methionine and acetylation of protein N termini. Different TMT settings and quantification methods were used for two versions of MaxQuant software. For MaxQuant (V1.2.2.5), the TMT tags on peptide N terminus (+229.1629 Da) and lysine residue were set as static modifications and protein quantification values were calculated with the improved Libra algorithm (12, 13). For MaxQuant software (V1.6.5.0), method type of reporter ion MS2 was used in the group-specific parameters module, and protein quantification values were calculated using the reporter ion MS2 method of isobaric labels in MaxQuant. Labeling efficiency was evaluated using the LC-MS/MS data of the first high-pH reversed-phase fraction of each TMT experiment, where TMT tags on lysine residues and peptide N termini were defined as variable modifications in MaxQuant. The efficiencies are 98.2% and 98.5% for the two labeling replicates of GV/GVBD/MII oocytes, respectively. And, the labeling efficiency for TMTpro 12-plex experiment (oocytes after MLN4924 treatment and Fbxo28 knockdown) is 94.2%.

### Bioinformatic Analysis

For oocyte maturation proteomics, significant differences in abundances between groups were statistically analyzed by an FDR-corrected *t* test. Volcano plots were constructed to better identify and display the differentially expressed proteins between groups. A dendrogram and heatmap were produced to depict the extent of protein expression similarity between the samples. For further annotation of differential proteins, we used Ensembl gene ID for bioinformatics analysis. To better characterize these differential proteins, we used the DAVIDs functional annotation tool (V6.8) to perform Kyoto Encyclopedia of Genes and Genomes enrichment analysis of the differential proteins. A gene ontology (GO) term or Kyoto Encyclopedia of Genes and Genomes pathway with FDR-q value <0.05 was considered significantly enriched. Pathway Studio (V6.00) software (Ariadne Genomics) was used to identify the GO enrichment analysis.

### Experimental Design and Statistical Rationale

Emerging evidence supports that maternal proteins stored in oocytes exert critical roles on meiotic maturation and embryo development. To identify the temporal proteome, the high-throughput proteomics analysis was performed on mouse oocytes during *in vivo* maturation (GV, GVBD, and MII). A total of four biological replicates were used for each stage (375 oocytes for each sample). In MLN4924 treatment and Fbxo28 knockdown experiments, a total of 1200 oocytes (dimethyl sulfoxide, 300; MLN4924, 300; siControl, 300; siFbxo28, 300; 3 replicates per treatment, each replicate containing 100 oocytes) were performed for proteomic profiles.

Statistical comparisons between two groups were performed with Student’s *t* test, and results are presented as means ± SD. Significance was accepted at *p*-value <0.05. For the TMT-based quantitative proteomics data, Student’s *t* test followed by Benjamini–Hochberg correction was used to control FDR. Each experiment was repeated at least three times, and the data shown in the figures result from one representative experiment unless specified. All analyses were performed using Prism v9.0 software (GraphPad).

## Results

### Proteomic Profile of Mouse Oocyte Maturation

**Fig. 1:**
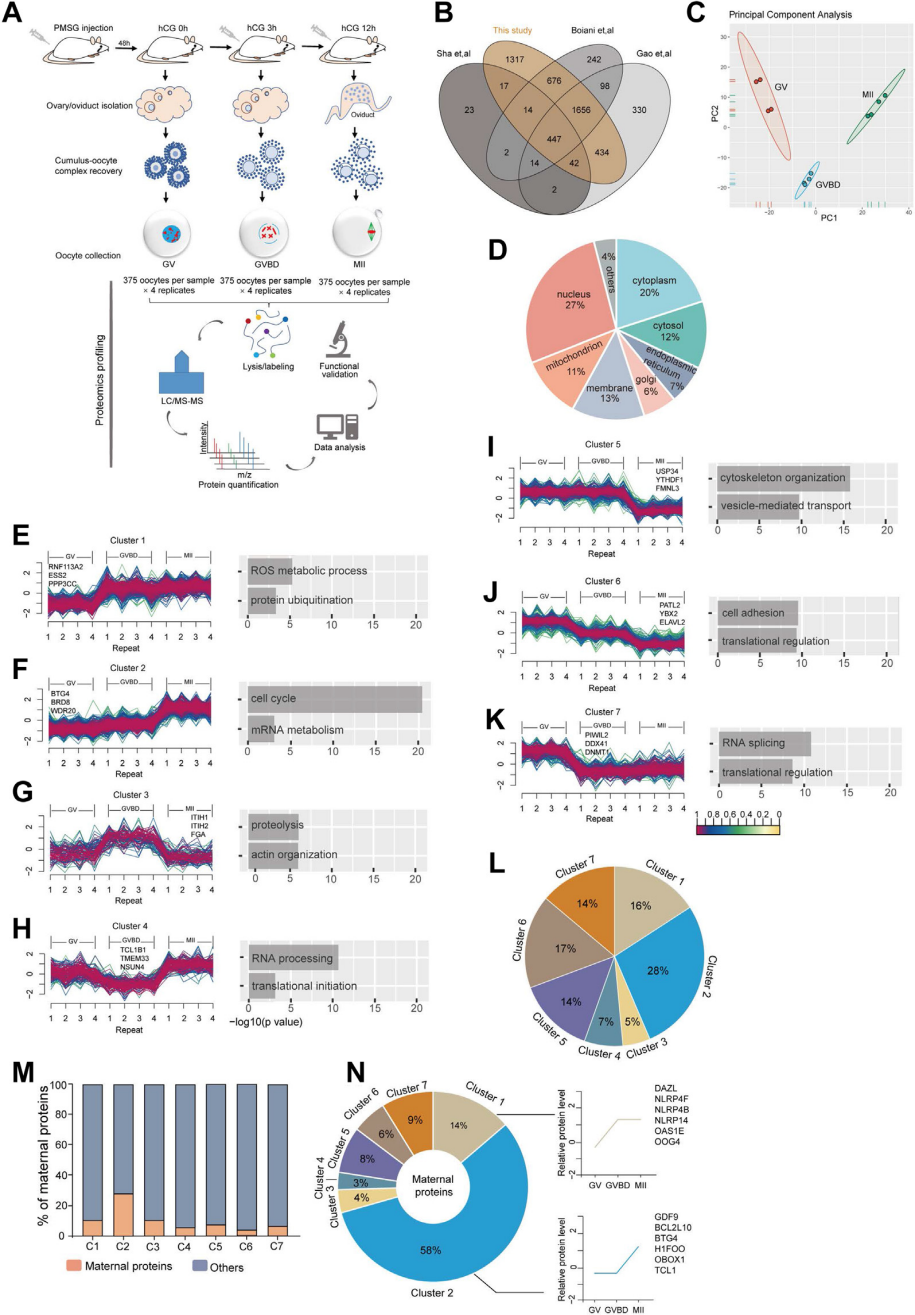
**Proteomic Profiling of Mouse Oocyte Maturation.***A*, schematic overview of the workflow for proteome profiling of mouse oocytes isolated at GV, GVBD, and MII stages. *B*, Venn diagram of protein numbers identified in different studies. *C*, principal component analysis of protein expression patterns of three different stages. *D*, diagram of proteins distribution among subcellular compartments. *E*–*K*, unsupervised c-means fuzzy clustering identified seven distinct profiles of proteins expression. The *x*-axis represents three developmental stages. The *y*-axis represents normalized intensity value and log2-transformed. Gene ontology analysis is shown on the right panel of each cluster. *L*, percentage of the protein number in each cluster. *M*, the yellow-colored bars indicate the percentage of maternal proteins in each cluster. *N*, pie chart shows the number of maternal proteins in each cluster. The *right line* chart represents the expression patterns of partial maternal proteins.

Using TMT-based LC-MS/MS, we systematically performed a quantitative proteomic analysis of GV, GVBD, and MII oocytes. The results showed a significant enrichment of categories related to metabolic pathways such as tricarboxylic acid cycle, and we systematically depicted the global metabolic patterns in oocytes as we previously published (4). In parallel, we conducted the bioinformatics analysis of the proteome and functional validation of critical factors to further reveal the proteomic dynamics in oocyte meiosis and molecular mechanisms for oocyte maturation. The experiment was conducted in biological quadruplicates with 375 oocytes per sample (Fig. 1*A*). In total, we successfully quantified 46,016 peptides and identified 4694 proteins (supplemental Table S1). More than 1300 proteins were uniquely found in this study as compared with published data (7, 8, 14), indicative of the high quality of our proteome profile (Fig. 1*B*). Principle component analysis revealed high quantitative reproducibility between biological replicates (Fig. 1*C*). Unsupervised hierarchical clustering showed that GV and GVBD were clustered together, and MII were further separated (supplemental Fig. S1*A*). Some well-characterized proteins known to be closely related to oocyte maturation (CYCLIN B1, BUB1B, BTG4, etc.) are significantly upregulated during meiosis (supplemental Fig. S1*B*), indicative of reliable data from the proteome. GO analysis showed that nuclear proteins represented the dominant proportion (27%), followed by cytoplasm proteins (20%) and membrane proteins (13%) (Fig. 1*D*).

Unsupervised clustering using fuzzy c-means algorithm (15) partitioned the proteomes into seven individual clusters with distinct temporal patterns, indicating the different expression kinetics (supplemental Table S2, Figs. 1, *E*–*L* and S1, *C*–*I*). Clusters 1 and 2 represent proteins that are upregulated, and clusters 5, 6, and 7 represent proteins that are downregulated; in contrast, clusters 3 and 4 represent proteins displaying a bimodal expression pattern. Specifically, cluster 2 consisted of 584 proteins that were highly expressed at the MII stage (Fig. 1, *F* and *L*), suggesting that more proteins increased through meiosis. Proteins related to cell cycle regulation and mRNA processing were frequently detected in cluster 2. For example, BTG4, a key maternal protein for mRNA decay in meiosis (16, 17), is a typical cluster 2 protein. The dramatic increase of BTG4 from GVBD to MII corresponds exactly to the beginning of maternal mRNA degradation from the GVBD stage (18). Consistent with the enriched term of translation regulation in cluster 6, translation repressors in germ cells (PATL2, YBX2, and ELAVL2) (19, 20) were downregulated (supplemental Fig. S1*H*). The proper degradation of these proteins may be responsible for the translation control during oocyte maturation. The meiotic maturation is closely associated with the dynamic changes in maternal proteins (proteins that are produced during oogenesis and required for oocyte maturation, fertilization, zygotic gene activation, and the early stages of embryogenesis) (21, 22). We noticed that the majority of maternal proteins are classified into cluster 1 (14%; i.e., DAZL, NLRP, and OOG4) and cluster 2 (58%; *i.e.*, GDF9 and BCL2L10), displaying a progressive increase in meiosis (Fig. 1, *M* and *N* and supplemental Table S3). The results above indicate that maternal proteins gradually accumulate during oocyte maturation and play key roles in subsequent embryonic development.

### Analysis of Differential Expression Proteins during Oocyte Maturation

**Fig. 2:**
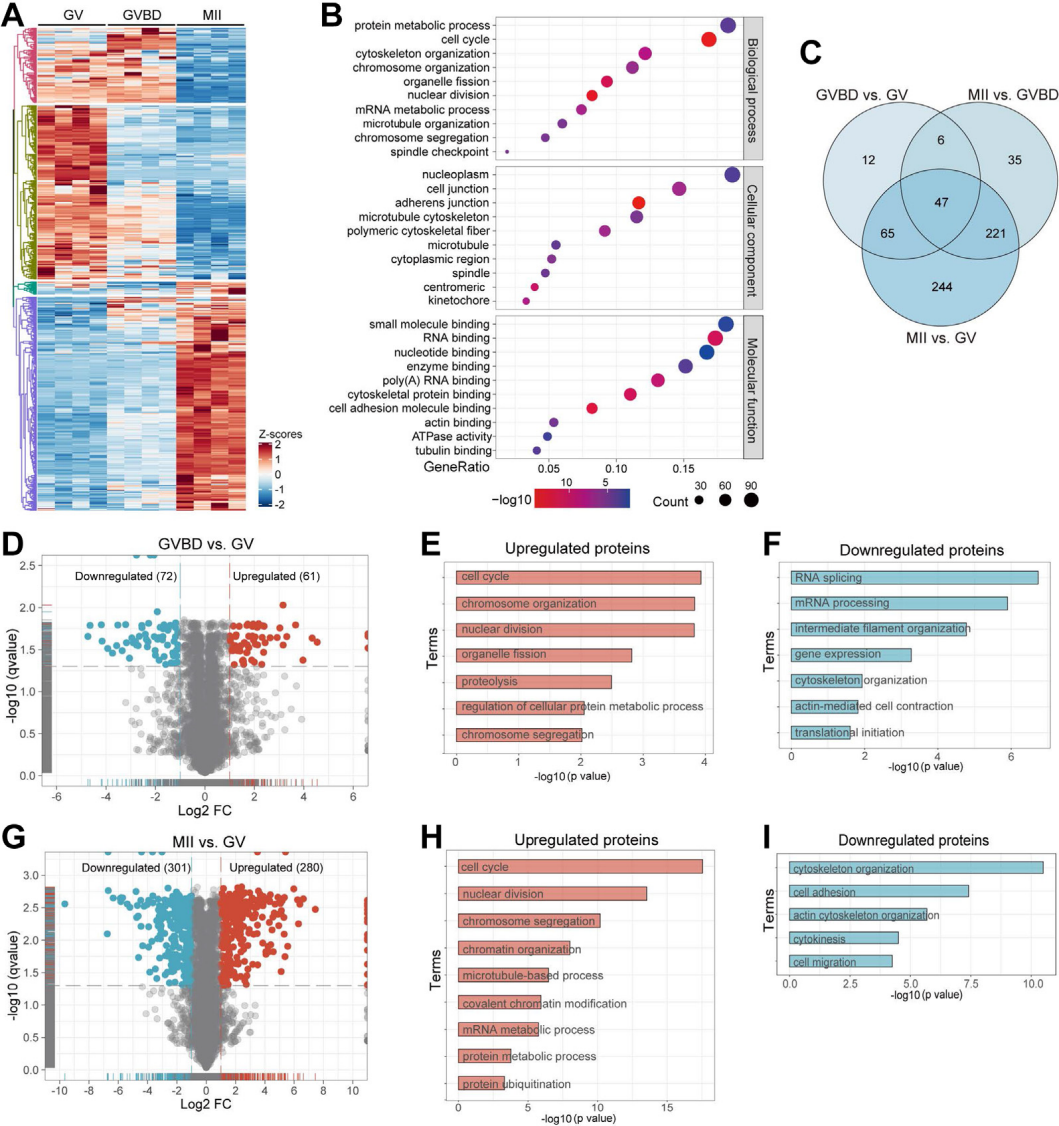
**Functional Analysis of Differentially Expressed Proteins.***A*, heatmap showing the expression profile of 634 differentially expressed proteins (DEPs, fold change>2, false discovery rate <0.05) during oocyte maturation. *B*, gene ontology (GO) enrichment analysis of DEPs was performed using the Pathway Studio (V6.00) software. *C*, Venn diagram showing the exclusive or shared DEPs between three different stages. *D*, volcano plot showing DEPs (downregulated, *blue*; upregulated, *red*) in GV and GVBD oocytes. (E-F) GO enrichment analysis of upregulated and downregulated DEPs in GV and GVBD oocytes. *G*, volcano plot showing DEPs (downregulated, *blue*; upregulated, *red*) in GV and MII oocytes. *H*–*I*, GO enrichment analysis of upregulated and downregulated DEPs in GV and MII oocytes.

To gain a better understanding of the meiotic maturation, differentially expressed proteins (DEPs) (fold change >2, FDR <0.05) were further analyzed. A total of 634 DEPs were identified as shown in Figure 2*A*. The GO terms were highly enriched in processes involved in protein metabolism, cell cycle, cell junction, and RNA processing (Fig. 2*B*). The numbers of exclusive or shared DEPs between distinct stages reflects the dramatic changes of proteomics feature between GV (immature state) and MII (mature state) oocytes (Fig. 2*C*). Furthermore, we evaluated the biological processes of DEPs between two distinct groups using GO and Gene Set Enrichment Analysis (Figs. 2, *D*–*I* and S2, *A*–*H*). Nuclear periphery proteins and keratins are predominantly downregulated in the GVBD stage (Figs. 2*F*, S2, *A* and *B*), consistent with the essential event of nuclear envelope breakdown during meiotic resumption (23, 24). A large number of proteins enriched in ubiquitination hydrolysis and mRNA metabolism are dramatically increased from the GV to MII stage (Figs. 2*H*, S2, *F* and *H*), reinforcing the notion that protein degradation and mRNA decay are active during oocyte maturation. Collectively, these data provide a broad resource of proteome dynamics during *in vivo* oocyte maturation. The findings highlight the importance of critical biological processes including cell cycle, ubiquitin hydrolysis, and mRNA metabolism in oocytes.

### Characterization of Cell Cycle Proteins during Oocyte Maturation

**Fig. 3:**
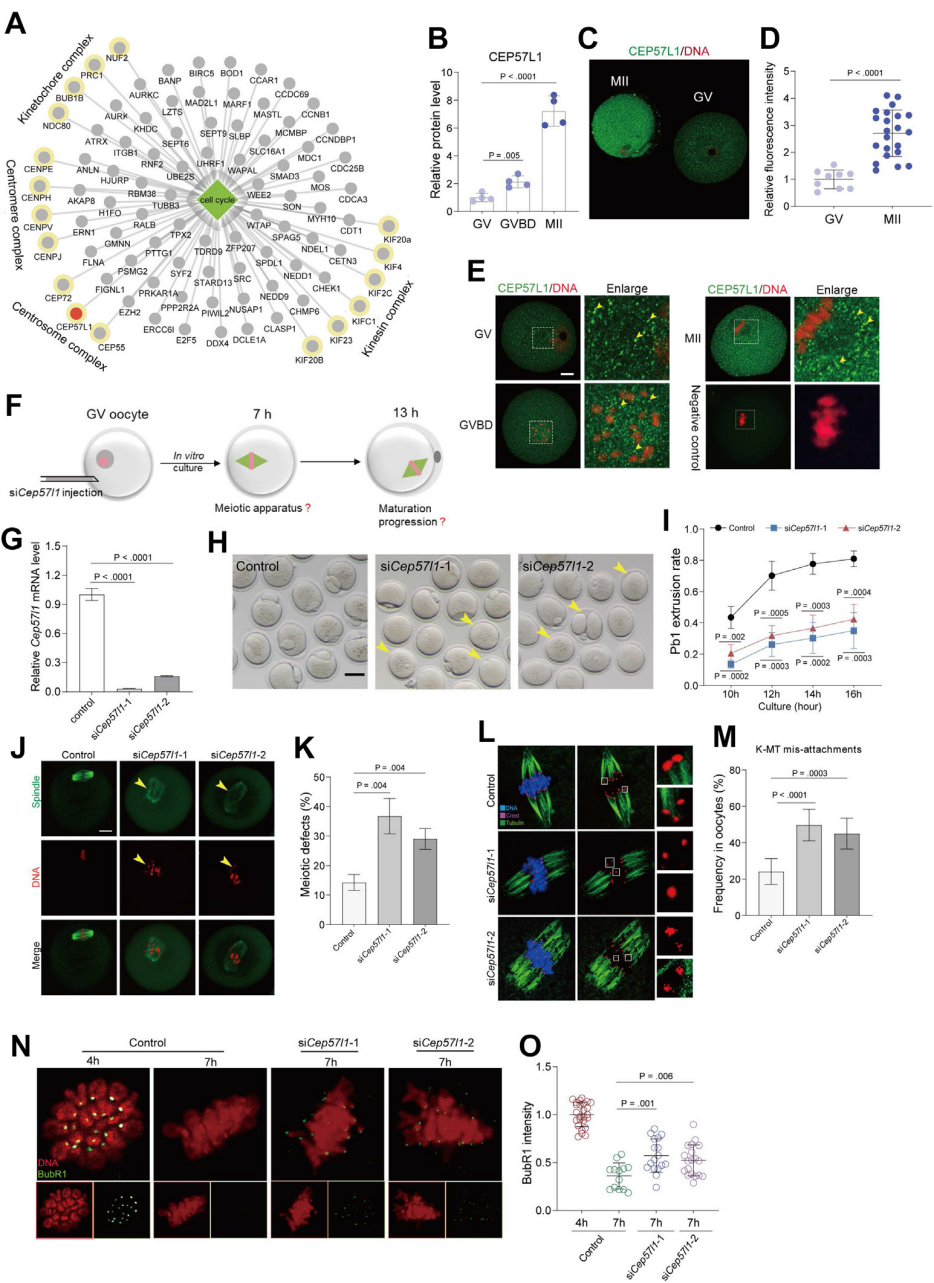
**CEP57L1 Regulates the Assembly of Meiotic Apparatus in Mouse Oocytes.***A*, gene ontology analysis of the differentially expressed proteins with enrichment in the regulation of cell cycle. The four complexes are marked with yellow circles. The red circle indicates CEP57L1. *B*, relative abundance of CEP57L1 in oocytes. *C* and *D*, relative fluorescence intensity of CEP57L1 in GV oocytes and MII oocytes. *E*, cellular distribution of CEP57L1 in oocytes at GV, GVBD, and MI stages. Arrowheads point to CEP57L1 signals. The scale bar represents 20 μm. *F*, schematic presentation of the experimental design to investigate the role of CEP57L1 in meiosis. *G*, knockdown efficiency of two *Cep57l1* siRNAs. *H*, representative images of oocytes from control and two si*Cep57l1* groups. Arrowheads point to oocytes that fail to extrude a polar body or oocytes with the abnormal polar body. The scale bar represents 50 μm. *I*, rate of Pb1 extrusion in control oocytes and two si*Cep57l1* groups. *J*, representative images of the spindle and chromosome morphology of MI oocytes stained with α-tubulin antibody (*green*) and with propidium iodide (*red*), respectively. *K*, quantification of control and two si*Cep57l1* groups with aberrant spindles. The scale bar represents 20 μm. *L*, representative images of kinetochore-microtubule (*K*–*M*) attachment in control and two si*Cep57l1* groups. Oocytes were costained with a-tubulin-FITC antibody (microtubules, *green*), CREST antibody (kinetochore, *purple*), and Hoechst 33342 (chromosomes, *blue*). *M*, quantitative analysis of the defective *K*–*M* attachment in the control group and two si*Cep57l1* groups. *N*, representative chromosome spread images of oocytes stained with BubR1 antibody (*green*) and propidium iodide (*red*) in the control group and two si*Cep57l1* groups. *O*, quantitative analysis of the BubR1 intensity in the control group and two si*Cep57l1* groups. Data are expressed as the mean ± SD from three independent experiments in which at least 100 oocytes were analyzed for each group. For statistical analysis, a two-tailed Student’s *t* test was used in all panels, compared with GV or control.

Meiosis is a complicated and vital process used to complete a cell division as well as asymmetric cytokinesis. Proteins related to cell cycle play critical roles in meiotic resumption and maturation. GO analysis of DEPs showed that the cell cycle process is the most enriched pathway among all terms (supplemental Fig. S1, *B* and *E*), and about 90 DEPs are found to be associated with this event (Fig. 3*A*). Most of these DEPs belong to four protein families including the centrosome complex, centromere complex, kinetochore complex, and kinesin complex (Figs. 3*A* and S3*A*), displaying the upregulated tendency from GV to MII stage (supplemental Fig. S3*B*). Of note, KIFC1, a kinesin family member, was recently demonstrated to be essential for microtubule stability in mouse oocytes and can rescue spindle instability in human oocytes (25, 26). The analysis discovered the involvement of cell cycle–related proteins in meiotic maturation, and among them CEP57L1 was selected for further functional investigation.

### CEP57L1 Promotes the Assembly of Meiotic Apparatus in Oocytes

Despite the loss of centrioles and the absence of classical centrosomes in mouse oocytes, centrosomal proteins and pericentriolar proteins are nonetheless abundantly expressed during meiotic division (27). Centrosomal protein 57 like protein 1 (CEP57L1), a paralog of CEP57, is conserved in vertebrates. CEP57 and CEP57L1 redundantly regulate centriole engagement during mitosis by recruiting the CEP63–CEP152 complex to the mother centrioles (28). Notably, *Cep57l1* mRNA is highly expressed in oocytes compared with other tissues (supplemental Fig. S3*C*), and CEP57L1 protein is dramatically accumulated during meiotic division (Fig. 3, *B*–*D*). Confocal imaging showed that CEP57L1 distributes throughout the oocytes and has spot-like concentration around chromosomes (Fig. 3*E*), indicating that CEP57L1 may be involved in regulating meiosis. To test this hypothesis, GV oocytes were microinjected with two interfering RNAs (siRNAs) targeting *Cep57l1* (si*Cep57l1*-1 and si*Cep57l1*-2) and then cultured for 14 h in the presence of milrinone (Fig. 3*F*), resulting in a substantial decrease in *Cep57l1* mRNA (Fig. 3*G*). Although si*Cep57l1* oocytes underwent GVBD normally, Pb1 extrusion (first polar body extrusion) was significantly reduced compared with controls after culture for 16 h (Figs. 3*H*, *I* and S3*D*), indicative of maturational defects. Meanwhile, the specific localization of CEP57L1 in oocytes prompted us to investigate whether CEP57L1 knockdown affects meiotic apparatus. Most control oocytes (approximately 86%) exhibited typical barrel-shaped spindles with well-aligned chromosomes at the equatorial plate. In contrast, CEP57L1 depletion resulted in the spindle disorganization and chromosomal congression failure (36% in the si*Cep57l1*-1 group; 28% in the si*Cep57l1*-2 group) (Fig. 3, *J* and *K*). During meiosis, chromosome movement requires dynamic coordination between microtubules and kinetochores. In line with this notion, CEP57L1 knockdown induced high frequency of the kinetochore-microtubule (K-MT) misattachments in MI stage oocytes (Fig. 3, *L* and *M*), resulting in the establishment of unstable chromosome biorientation. Spindle assembly checkpoint (SAC), a surveillance pathway in mammalian cells, is required for monitoring and responding to K-T attachment. Budding uninhibited by benzimidazole-related 1 (BubR1), an integral component of the SAC, is commonly used to evaluate the status of SAC (29). In normal oocytes, BubR1 localizes to kinetochores at prometaphase I stage but loses this localization pattern when properly attached to chromosomes at metaphase I stage. However, the BubR1 signal on the kinetochores was markedly increased in CEP57L1-depleted oocytes (Fig. 3, *N*–*O*), indicating that the defective K-MT attachments contributes to abnormal SAC activation.

CEP57, a paralog of the CEP57L1, is another centrosomal protein essential for centriole engagement. CEP57 was undetectable in the proteomic profile probably due to its low mRNA expression compared with *Ce*p57l1 (supplemental Fig. S3*E*). The amino acid sequences and spatial structure of CEP57 and CEP57L1 are similar (30) (supplemental Figs. S3*F* and 3*G*). On the other hand, the structural differences of the C-terminal domain may cause functional variations between CEP57 and CEP57L1. To test whether CEP57 also participates in the regulation of meiotic maturation, we performed knockdown experiments by injecting *Cep57*-specific siRNA into fully grown oocytes. The *Cep57* mRNA level was effectively reduced as shown by qRT-PCR (supplemental Fig. S3*H*). However, CEP57-depleted oocytes presented the normal phenotype, implying that it is dispensable for the meiotic maturation (supplemental Figs. S3*I* and 3*J*). Together, these results indicate that CEP57L1, but not CEP57, promotes the assembly of meiotic apparatus in mouse oocytes.

### Expression Patterns of Epigenetic Modifiers during Oocyte Maturation

**Fig. 4:**
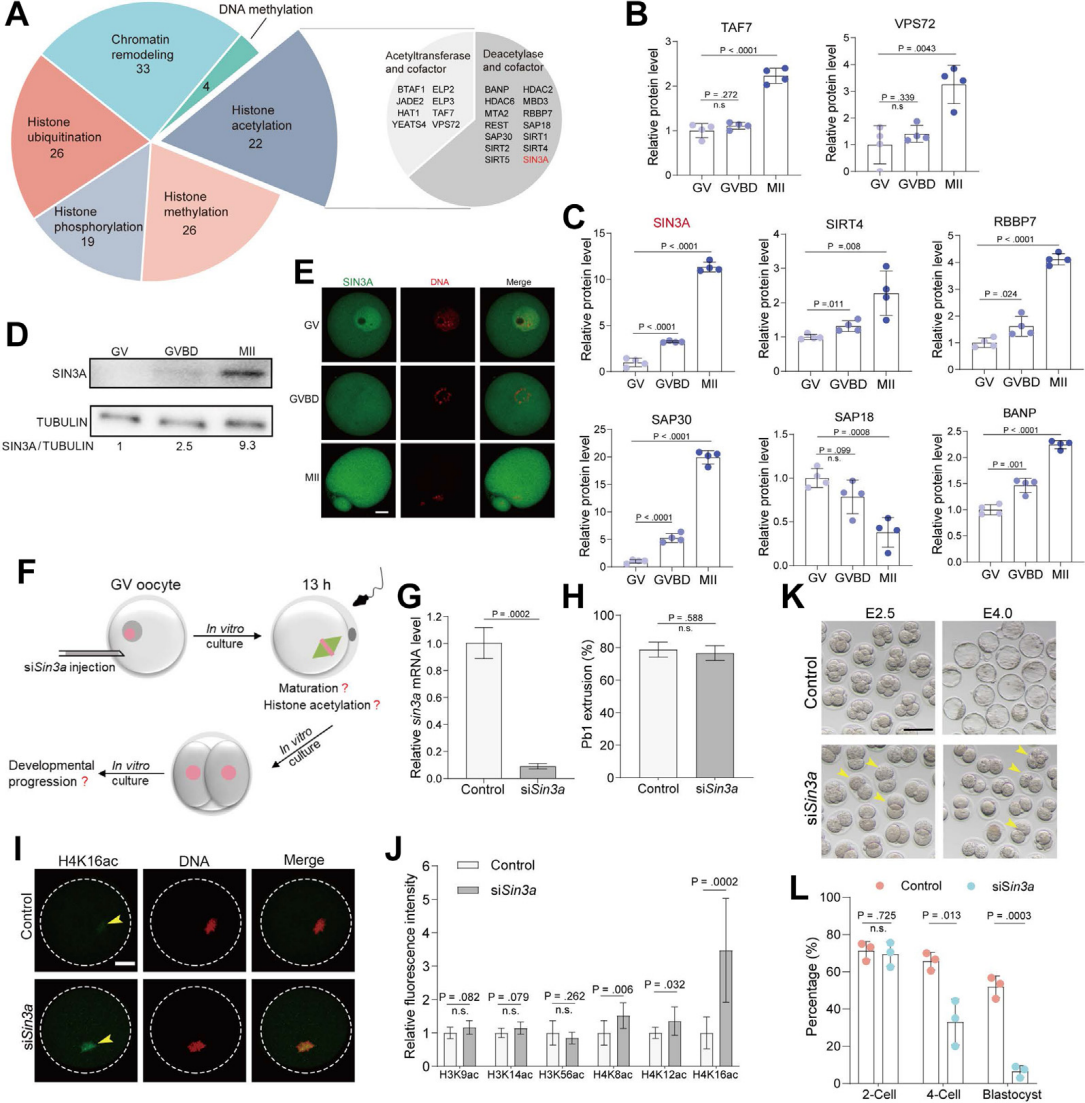
**SIN3A Regulates Histone Deacetylation in Mouse Oocytes.***A*, pie plot showing the epigenetic modulators in oocytes. Histone acetylases, deacetylases, and their cofactors are shown in the right pie plot. *B*, relative abundance of two histone acetyltransferases at GV, GVBD, and MII stages. *C*, relative abundance of six histone deacetylases in oocytes of GV, GVBD, and MII stages. *D*, SIN3A protein expression measured by Western blot at GV, GVBD, and MII stages. *E*, immunostaining analysis of SIN3A distribution in GV, GVBD, and MII oocytes. DNA was stained with propidium iodide. The scale bar represents 20 μm. *F*, schematic presentation of the experimental design to investigate the role of SIN3A during oocyte development. *G*, the efficiency of *Sin3a* siRNA knockdown was determined by RT-qPCR. *H*, rate of Pb1 extrusion in control oocytes and si*Sin3a* oocytes. *I*, representative images of H4K16ac fluorescence in control oocytes and si*Sin3a* oocytes. The scale bar represents 20 μm. *J*, quantitative analysis of fluorescence intensity of acetylated histones. *K*, bright-field images of E2.5 embryos and E4.0 embryos derived from control and si*Sin3a* oocytes. The scale bar represents 100 μm. *L*, percentage analysis of development rate in two-cell embryos, four-cell embryos, and blastocysts derived from control and si*Sin3a* oocytes. Data are expressed as the mean ± SD from three independent experiments in which at least 100 oocytes were analyzed for each group. For statistical analysis, a two-tailed Student’s *t* test was used in all panels, compared with GV or control. n.s., not significant.

Oocyte histones are present in a variety of posttranslational modifications, including acetylation, methylation, phosphorylation, and ubiquitination. Epigenetic modifiers play different but essential roles in oocyte meiosis (31, 32, 33, 34). For example, inadequate histone deacetylation during oocyte maturation leads to aneuploidy and abnormal embryo development in mice (35). Here, our proteomic profiling identified 97 epigenetic regulators and 33 chromatin remodelers in meiotic oocytes (Fig. 4*A* and supplemental Table S4). In particular, we found that most modifiers are upregulated (supplemental Fig. S4*A*), implying the dramatic epigenome remodeling during meiotic maturation. In addition, we identified 26 histone ubiquitination-associated factors that are responsible for histone ubiquitination and degradation (Fig. 4*A*). In line with this observation, different isoforms of linker histone H1 (HISTH1A, HISTH1B, HISTH1C, HISTH1D, and HISTH1T) are almost degraded from GV to MII stage (supplemental Fig. S4*B*).

We next focused on the enzymes and cofactors related to histone acetylation (Fig. 4, *A*–*C*). It is worth noting that five of six these proteins increased significantly from GV to MII stages, consistent with the extensive histone deacetylation during this process (36, 37). Of them, SIN3A is of great interest to us because (i) its protein accumulation is markedly elevated in meiosis (Fig. 4*C*; GVBD/GV: ∼3-fold and MII/GV: ∼11-fold); (ii) it is a highly conserved protein with emerging roles in deacetylation (38).

### SIN3A Is Essential for Histone Deacetylation in Oocytes

SIN3A is a core scaffolding protein of SIN3–HDAC complexes involved in histone deacetylation, transcriptional repression, and DNA repair (39). It has been reported that maternal SIN3A is required for morula-to-blastocyst transition in mouse embryos (40). Consistent with the proteomic data, immunoblotting showed that SIN3A protein expression is gradually increased during oocyte maturation (Fig. 4*D*). SIN3A was predominantly present in the nucleus of GV oocytes and then distributed in the cytoplasm of MII oocytes (Fig. 4*E*). Next, we assessed the effects of SIN3A depletion on the status of histones acetylation and developmental potential of oocyte (Fig. 4*F*). Interestingly, maturation progress was not disrupted in SIN3A-depleted oocytes (Figs. 4, *G*, *H* and S4*C*). We then examined global changes in acetylation levels of six lysine residues on histone H3 and H4 during meiosis (supplemental Fig. S4*D*). The acetylation levels of all lysine residues were remarkably decreased from GV to MII stage (supplemental Fig. S4*E*). In sharp contrast, the levels of H4K8ac, H4K12ac, and H4K16ac were significantly elevated in metaphase oocytes when SIN3A was knocked down (Figs. 4, *I*, *J* and S4*F*). The results indicate that SIN3A participates in the control of histone deacetylation. Deficient deacetylation in oocytes could affect developmental progression of early embryos (35). Therefore, we further evaluated the developmental potential of oocytes with SIN3A depletion. Importantly, after fertilization, about 30% of embryos derived from si*Sin3a* injected–oocytes developed to four-cell stage on day 2.5, which is significantly lower than that in controls (65%), as illustrated in Figure 4, *K* and *L*. In addition, only limited fraction of embryos in the SIN3A-depleted group developed to blastocysts. Inadequate histones deacetylation induced by SIN3A depletion may be responsible for the developmental arrest of early embryos. Altogether, we identified SIN3A as a novel effector modulating histone deacetylation during mouse oocyte maturation.

### Active mRNA Metabolism during Maturation

**Fig. 5:**
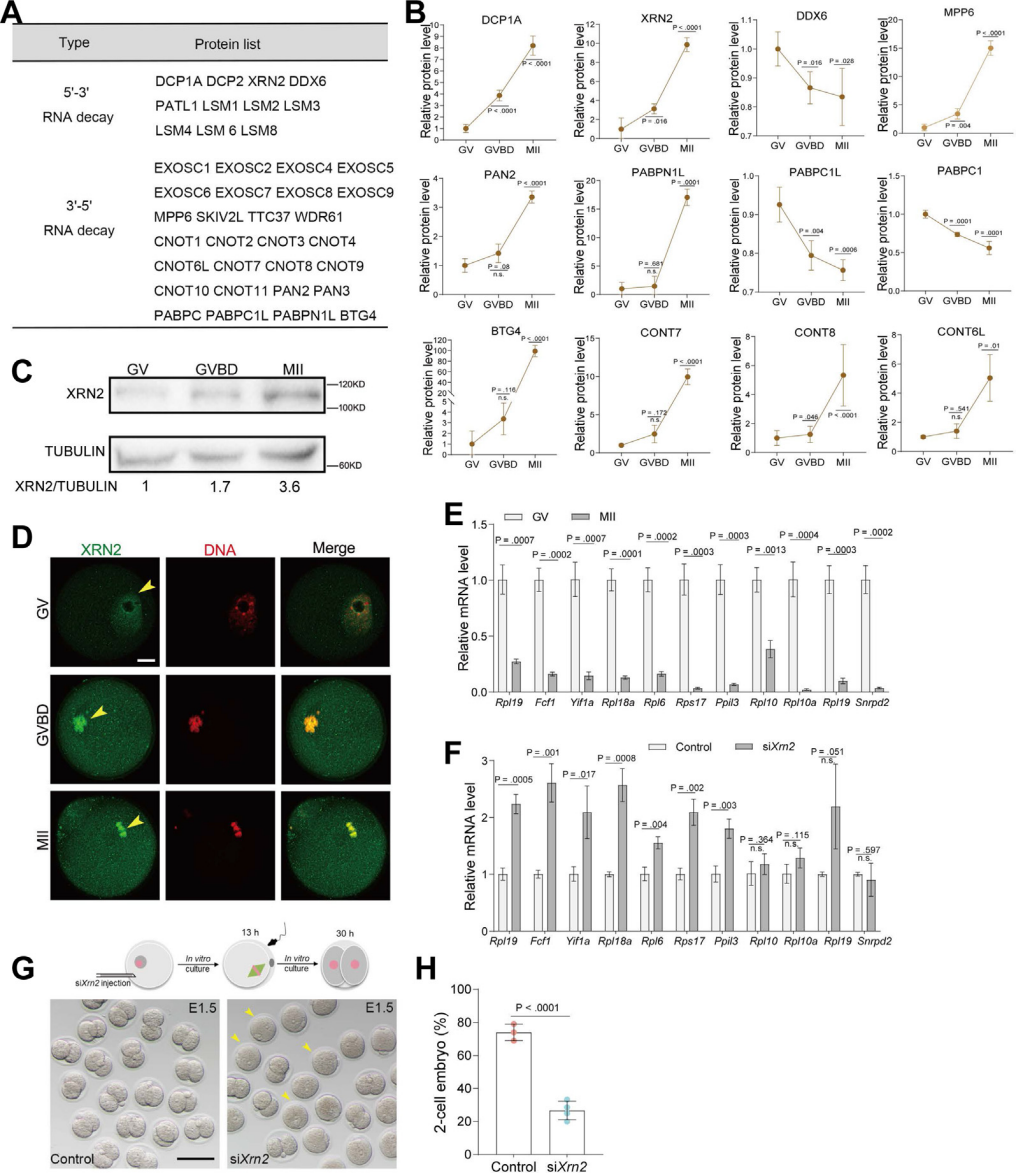
**XRN2 Degrades Maternal mRNA in Oocytes.***A*, proteins associated with 5′-3′ RNA decay and 3′-5′ RNA decay. *B*, differentially expressed proteins associated with 5′ mRNA decay pathway and 3′ mRNA decay pathway. *C*, XRN2 protein expression in oocytes collected *in vivo*. *D*, immunostaining analysis of XRN2 distribution in GV, GVBD, and MII oocytes. DNA was stained with propidium iodide. Arrowheads point to XRN2 signals. The scale bar represents 20 μm. *E*, RT-qPCR confirmed the expression levels of the representative mRNA degraded from the 5′ end between GV oocytes and MII oocytes. *F*, quantification of the selected mRNA by RT-qPCR to assess the effect of XRN2 knockdown. *G*, bright-field images of E1.5 embryos derived from control and XRN2 knockdown oocytes. The scale bar represents 100 μm. *H*, percentage analysis of development rate in two-cell embryos derived from control and SIN3A-depleted oocytes. Data are expressed as the mean ± SD from three independent experiments in which at least 100 oocytes were analyzed for each group. For statistical analysis, a two-tailed Student’s *t* test was used in all panels, compared with GV or control. n.s., not significant.

It has been widely accepted that maternal mRNA decay is essential for maternal-to-zygotic transition (41). mRNA degradation mediated by Poly(A) shortening in oocytes, named 3′ mRNA decay pathway, has been well studied (42). In eukaryotic cells, the 5′ cap not only regulates translation but also controls mRNA stability. Maternally recruited DCP1A and DCP2, two decapping players, are required for decapping maternal mRNA and transiting from mRNA stability to instability during meiotic maturation (43). Decapped mRNA will be exposed to exonucleases that rapidly degrade mRNAs from the 5′ end, called 5′ mRNA decay pathway. To date, little is known about what exonucleases contribute to maternal mRNA degradation from the 5' end. To dissect these two distinct pathways in oocytes, identified proteins related to mRNA metabolism were categorized based on the 3’ mRNA decay and 5′ mRNA decay pathways (Figs. 5*A*, S5, *A*, and *B*). Key factors involved in mRNA decay were accumulated in mature oocytes (Fig. 5*B*). In particular, XRN2, a nuclear exoribonuclease that selectively targets decapped mRNA (supplemental Fig. S5*C*) (44), significantly upregulated during maturation (Fig. 5*C*). XRN1, another key exoribonuclease, was not identified in our proteome analysis, possibly due to the limited transcript level of the corresponding gene (supplemental Fig. S5*D*) (18). The enrichment of XRN2 in oocytes prompted us to investigate its functional significance.

With the resumption of meiosis, the oocyte nucleus disappears triggered by germinal vesicle breakdown. XRN2 redistributes in oocyte cytoplasm after being released from the nucleus; however, it still predominantly localizes on chromatin (Fig. 5*D*). The chromatin localization of XRN2 may be caused by its particular involvement in transcription termination (supplemental Fig. S5*C*) (45). The cytoplasm localization prompted us to check whether cumulative XRN2 participates in the degradation of maternal mRNA. Several maternal mRNAs decapped by DCP1A and DCP2 were selected to quantify their expression levels (Fig. 5*E*) (43). Their mRNA abundance was significantly upregulated in MII oocytes with XRN2 depletion (Fig. 5*F*), indicative of the insufficient degradation. Simultaneously, XRN2 was knocked down to check its effects on oocyte developmental competence. As shown in supplemental Fig. S5, *E* and *F*, XRN2 loss appeared not to have evident effect on execution of meiotic maturation, albeit a slight decrease in Pb1 extrusion was detected. Abnormal degradation of maternal mRNA leads to failure of zygotic genome activation and subsequent embryo development (16). Next, these oocytes were injected with control siRNA or si*Xrn2* and then cultured *in vitro* after fertilization. Of note, the majority of those embryos in the XRN2-depleted group were arrested at the one-cell stage (Fig. 5, *G* and *H*). The findings emphasize the potential importance of degrading maternal transcripts through the 5′ mRNA decay pathway in mouse oocytes.

### Ubiquitination Pathways during Oocyte Maturation

**Fig. 6:**
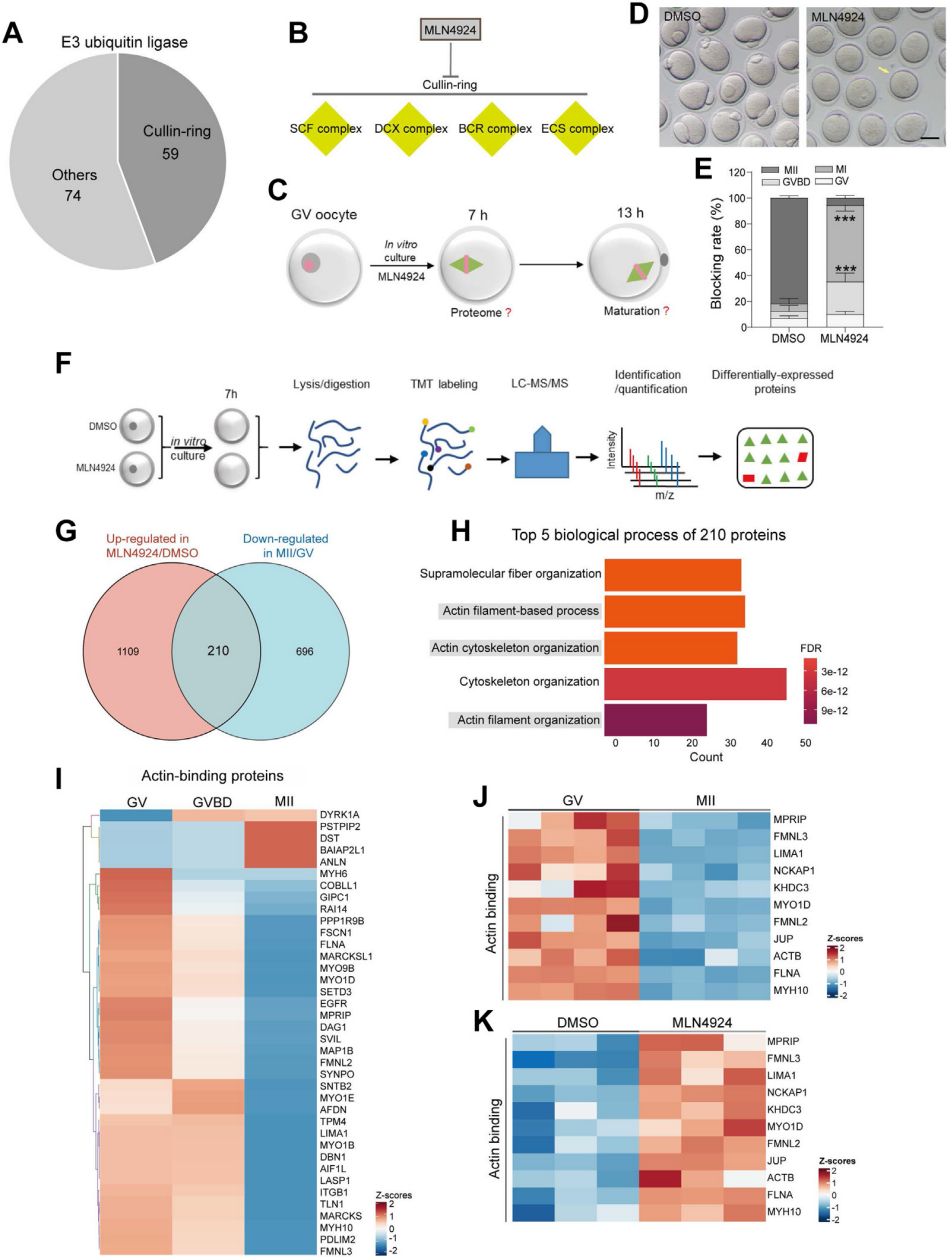
**CRLs modulate oocyte maturation by Controlling the Degradation of Actin-Related Proteins.***A*, pie plot showing the Cullin-RING ubiquitin ligases (CRLs) and other ligases identified in the proteome. *B*, schematic diagram showing that four complexes of CRLs are inhibited by MLN4924. *C*, schematic presentation of the experimental design to investigate the effect of CRLs inhibition on oocyte maturation. *D*, bright-field images of oocytes treated with dimethyl sulfoxide (DMSO) and MLN4924. The scale bar represents 100 μm. *E*, percentage of four meiotic stages (GV, GVBD, MI, and MII) in oocytes treated with DMSO and MLN4924. *F*, overview of a proteomic method for identifying differential proteins. *G*, Venn diagram showing the exclusive or shared upregulated proteins in oocytes treated with DMSO and MLN4924. *H*, GO enrichment analysis. The bar plot depicts the top five enriched biological processes of 210 common proteins shown in Fig. 6*G*. *I*, heatmap showing the differentially expressed proteins related to actin binding. *J* and *K*, heatmap showing the expression of partial proteins related to actin organization. Data are expressed as the mean ± SD from three independent experiments in which at least 100 oocytes were analyzed for each group. For statistical analysis, a two-tailed Student’s *t* test was used in all panels, compared with GV or control. ∗∗∗*p* < 0.001.

Meiotic maturation necessitates timely degradation of particular proteins stored in the ooplasm (46). Several factors correlated with the degradation of maternal proteins have been reported previously, especially the anaphase-promoting complex (APC) (47). However, the ubiquitin proteasome system, the primary regulatory process for protein degradation, remains to be fully understood in oocytes. In mammalian cells, there are three different sets of enzymes (E1, E2, and E3) that shuttle Ub and eventually link Ub to substrates. The substrates tagged by Ub are degraded indiscriminately by 26S proteasome (supplemental Fig. S6*A*) (48). Numerous E3 ligases, particularly Cullin-RING ubiquitin ligases (CRLs, the prototypical multisubunit complex), are identified from our proteome data (Figs. 6*A* and S6, *B*–*E*).

#### CRLs Modulate oocyte maturation by Controlling the Degradation of Actin-Related Proteins

Cullin subunits serve as the central assembly scaffold of CRLs. NEDD8-activating enzyme 1 (NAE1) modulates the activity of CRLs through neddylation of cullin proteins (49). To explore the role of CRLs in meiosis, we first employed MLN4924 to disrupt NAE1 function (Fig. 6, *B* and *C*). Fully grown oocytes were treated with 1 μM MLN4924 and cultured for 13 h. As shown in Figure 6, *D*–*E*, most oocytes were blocked at the MI stage following CRLs inhibition. To elucidate the potential mechanisms, a comparative proteomic analysis was performed (Fig. 6*F* and supplemental Table S5). Since CRLs primarily mediate protein degradation, we analyzed the upregulated proteins (1319) in MLN4924-treated oocytes (group 1; FDR = 0.05, Fig. 6*G*). Through the combined analysis of the downregulated proteins during normal oocyte maturation (group 2; FDR = 0.05), we found that 210 proteins are shared between two groups (Fig. 6*G*). Degradation of these proteins is under the control of the CRLs in a direct or indirect way. Moreover, GO analysis revealed that cytoskeletal organization, specifically the actin filament–based process, is severely disturbed when CRL is inhibited (Fig. 6*H*). Consistently, many actin-binding proteins were downregulated during normal meiotic maturation (Fig. 6*I*); by contrast, the degradation trend of these proteins was prevented following MLN4924 treatment (Fig. 6, *J* and *K*). The results suggest that CRLs regulate oocyte maturation by, at least in part, controlling the degradation of actin-related proteins.

#### FBXO28 Regulates Meiotic maturation via Degrading UBAP1

**Fig. 7:**
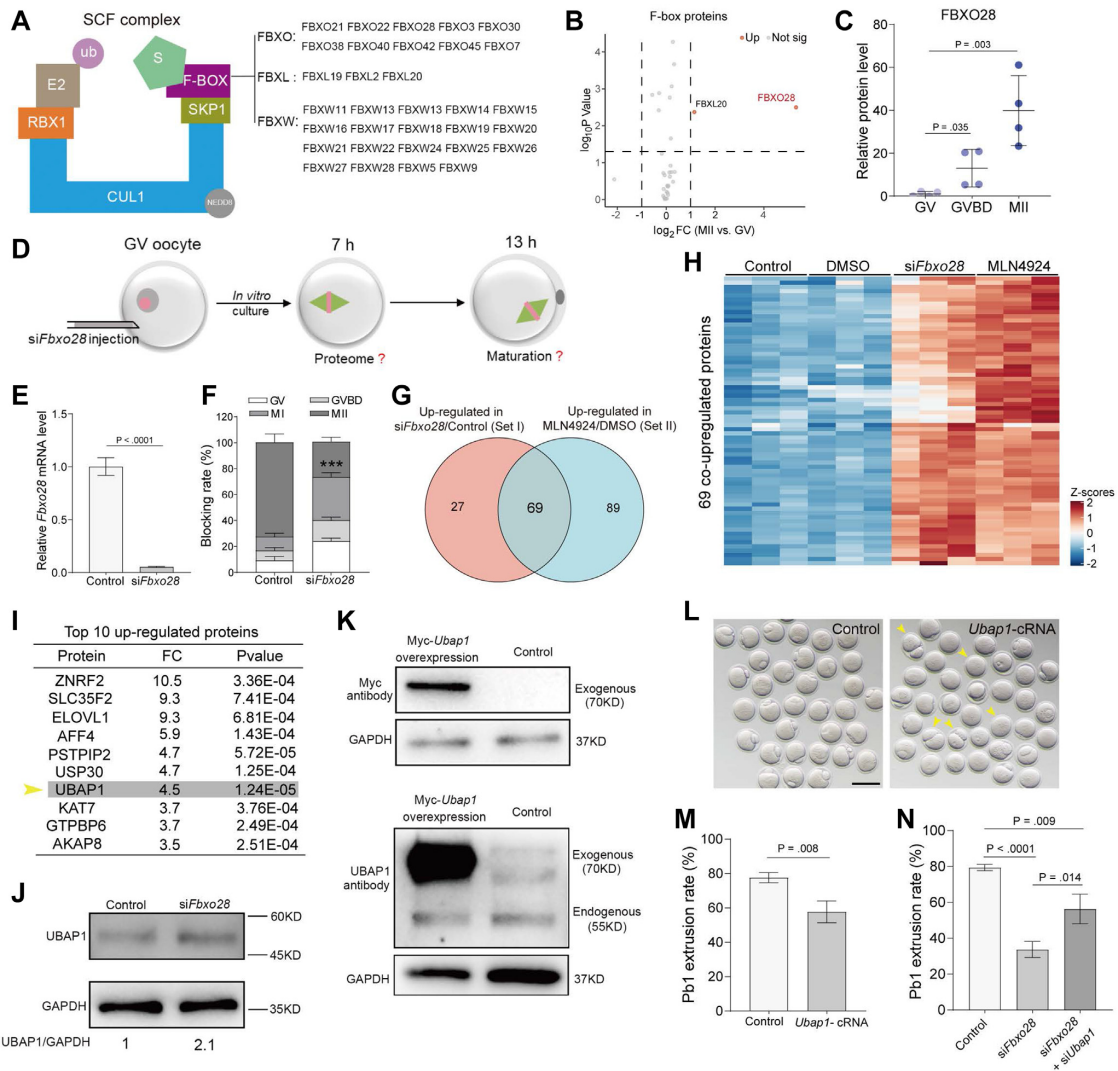
**FBXO28 Is A Key F-box Protein for SCF Activity.***A*, schematic diagram showing the structure of SCF complex. F-BOX proteins detected in proteome are shown in the right panel. *B*, volcano plot showing the relative abundance of F-BOX proteins between GV oocytes and MII oocytes. Differential F-BOX proteins (fold change >2) are indicated by *red dots*. *C*, relative FBXO28 protein level in oocytes. *D*, schematic presentation of the experimental design to investigate the role of FBXO28 during oocyte maturation. *E*, knockdown efficiency of *Fbxo28* siRNA. *F*, percentage of four meiotic stages (GV, GVBD, MI, and MII) in oocytes injected with control siRNA and si*Fbxo28*. *G*, Venn diagram showing the exclusive or shared upregulated proteins in oocytes of MLN4924 treated group (set I) and FBXO28-knockdown group (set II). *H*, heatmap showing the 69 common proteins upregulated in both the control group and the FBXO28-depletion group. *I*, *top* 10 upregulated proteins in oocytes with FBXO28 depletion. *J*, UBAP1 protein expression in control and FBXO28-depletion oocytes. *K*, immunoblotting results show the overexpression (OE) of exogenous UBAP1 protein in oocytes. *L*, bright-field images of control and UBAP1-OE oocytes. *Arrowheads* point to oocytes that fail to extrude a polar body. The scale bar represents 100 μm. *M*, quantitative analysis of Pb1 extrusion rate in control and UBAP1-OE oocytes. *N*, Pb1 extrusion rates of control, FBXO28-depletion, and FBXO28/UBAP1 double depletion oocytes. Data are expressed as the mean ± SD from three independent experiments in which at least 100 oocytes were analyzed for each group. For statistical analysis, a two-tailed Student’s *t* test was used in all panels, compared with GV or control. ∗∗∗*p* < 0.001.

CRLs are divided into four families (SCF, BCR, DCX, and ECS complex) based on diverse components, especially cullin proteins (supplemental Fig. S7*A*). Numerous proteins related to the SCF (Skp1–Cullin1–F-box) complex are identified in mouse oocytes based on our proteomic data (supplemental Figs. S7*B* and S6*E*). As the scaffold of SCF, Cullin 1 (Cul1) mRNA is highly expressed in oocytes (supplemental Fig. S7*C*). Its protein level is also upregulated during oocyte maturation (supplemental Fig. S7*D*). SCF generally recognizes well-defined substrates via F-BOX proteins. A total of 32 F-BOX proteins were identified in oocyte proteome, including 10 FBXOs, 3 FBXLs, and 19 FBXWs (Fig. 7*A*). Of them, we noticed that FBXO28 is markedly elevated from the GV to MII stage (Fig. 7, *B* and *C*). Furthermore, the *Fbxo28* transcript is uniquely expressed in oocytes relative to other tissues (supplemental Fig. S7, *E* and *F*) (50). These findings indicate that the SKP1–CUL1–FBXO28 (SCF^FBXO28^) complex may be functional in oocytes. To check the potential role of FBXO28 in oocyte development, endogenous *Fbxo28* mRNA was efficiently depleted by siRNA injection (Fig. 7, *D*–*E*). As shown in Figure 7*F*, FBXO28-depleted oocytes were arrested at the MI stage. Next, we performed a comparative proteomic analysis of oocytes cultured for 7 h to explore the substrates of FBXO28 (supplemental Fig. S8*A*), and the expression of 96 proteins were found to be significantly increased (fold change>1.5, refer to set I; supplemental Table S5). It is worth noting that 69 proteins were also upregulated following MLN4924 treatment (fold change>1.5, refer to set II) (Fig. 7, *G* and *H*). Considering that the comparable phenotypes present in FBXO28-depelted oocytes and CRLs-inhibited oocytes, we reason that FBXO28 might work as a vital regulator in CRLs for proteins degradation during oocyte maturation.

UBAP1, a component of the endosome-specific ESCRT-I complex, is involved in sorting of endocytic ubiquitinated cargos into multivesicular bodies, which are essential for the maintenance of cellular homeostasis (51). Its protein level increased about 4.5-fold and 2.1-fold in FBXO28-depleted oocytes confirmed by proteome and immunoblotting, respectively (Fig. 7, *I* and *J*). UBAP1 was also among the top 10 upregulated proteins in both groups (set I and set II) (supplemental Fig. S1, *B*–*D*), suggesting that it was degraded dramatically by the SCF^FBXO28^ complex. UBAP1 overexpression significantly disrupted oocyte maturation (Fig. 7, *K*–*M*), whereas knockdown had no effect on polar body emission (supplemental Fig. S8, *E* and *F*), suggesting that excess but not loss of UBAP1 could result in maturation arrest. Considering that FBXO28 depletion induced UBAP1 accumulation in oocytes, we therefore examined whether UBAP1 reduction could rescue the defective phenotypes of si*FBXO28* oocytes. As shown in Figure 7*N*, UBAP1 knockdown partially promoted the polar body extrusion of FBXO28-depleted oocytes. Altogether, these data suggest that FBXO28, as a key regulator in CRLs, controls oocyte maturation through the degradation of UBAP1.

## Discussion

Mammalian oocytes contain an abundance of proteins required for subsequent developmental processes such as meiosis, fertilization, and embryogenesis. Owing to the scarcity of experimental material, it is difficult to execute a comprehensive proteome profile of mouse oocytes. In the present study, we utilized the optimized LC-MS/MS method to systematically delineate the proteomic dynamics during mouse oocyte *in vivo* maturation. We identified 4694 proteins, and 634 of them were significantly changed across multiple stages, suggesting a large scale of proteome remodeling. This study not only identified more proteins in oocytes but also provides a comprehensive analysis of the critical biological events during meiosis.

**Fig. 8:**
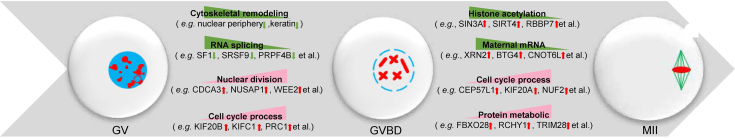
**Summary of Proteomics Profile in Meiosis.** From the GV stage to the GVBD stage, proteins of the nuclear periphery and keratins are downregulated, while CDCA3 and NUSAP1, etc. are upregulated. Dynamic changes in these proteins are associated with nuclear envelope breakdown. From the GV stage to the MII stage, the upregulation of deacetylases and exonucleases resulted in maturation-related histone deacetylation and maternal mRNA degradation, the upregulation of proteins related to the cell cycle results in meiotic progression. Meanwhile, key proteins and pathways, such as FBXO28 and CRLs, respectively, regulate oocytes maturation by targeting and degrading specific substrates

In the present work, we studied the proteome dynamics to illustrate the functions of critical proteins and pathways for oocyte maturation, as shown in Figure 8, including 1) a progressive increase in the levels of proteins related to cell cycle regulation, such as centrosomal proteins and centromeric proteins; 2) a sharp decline in histone acetylation with deacetylases increase; 3) maternal mRNA decay with exoribonucleases upregulation; and 4) protein degradation with active SCF^FBXO28^ complex in mouse oocytes.

### Histone Deacetylation during Meiosis

Global histone deacetylation is thought to be a prominent mechanism that takes place in meiosis and is essential for regulating proper chromosome condensation and chromosome segregation (52). Several key factors for regulating histone deacetylation have been identified, such as RBBP4, RBBP7, and HDAC2 (53, 54, 55). Here, we not only discovered a large number of histone deacetylases in mouse oocytes but also demonstrated that SIN3A is a novel regulator responsible for deacetylation of H4K8, H4K12, and H4K16 (Fig. 4). Zhao *et al.* reported that the amount of H3K27ac and H4K5ac was obviously improved upon SIN3A knockdown in mouse morula stage (38). Surprisingly, Jimenez, *et al.* observed the hypoacetylation in three histone markers of H3K18ac, H4K8ac, and H4K12ac when SIN3A was inhibited in mouse two-cell embryos (40). Several reasons may explain the discrepancy between these studies. At different stages, SIN3A may have distinct interacting proteins, which could lead to functional variability. Meanwhile, the expression pattern of SIN3A in oocytes and embryos is also different. The relative amount of SIN3A protein is increased from the GV to MII stage and then experiences a dramatic loss by the two-cell embryo stage (40). Regardless, the exact mechanism as to the differential activity of SIN3A still needs further exploration. These results indicate that SIN3A-mediated global histone deacetylation is important to establish the appropriate epigenetic marks for oocytes and early embryos.

### mRNA Decay in Oocytes

Our proteomic data quantified extensive proteins related to mRNA poly(A) deadenylation, such as the well-known factors BTG4, PABPN1L, and CONT6L (16, 56, 57). Although maternal mRNA decay from 3′ poly(A) has been well characterized (58), another mechanism essential for degrading mRNA from the 5′ ends is poorly understood. To better characterize this pathway, we performed a global search of the proteomics data to identify exoribonucleases for 5′ mRNA degradation. A total of 11 validated hits were identified, while most hits are subunits of RNA-binding Like-SM (LSM) complexes (*e.g.*, LSM1, LSM2, and LSM3) (Fig. 5). Here, we noted that XRN2 exoribonuclease may participate in the degradation of maternal mRNA. Previous work has demonstrated that XRN2 selectively contributes to silencing of a subset of H3K27me3-marked through posttranscriptional RNA decay (59). Whether the gene locus of maternal mRNAs degraded by XRN2 is associated with the H3K27me3 marker needs further investigation. The results remind us that it is necessary to explore the relationship between maternal mRNAs degradation in meiosis and the epigenetic marks (histone or DNA modifications) at the corresponding gene locus. It is commonly accepted that fully grown oocytes are transcriptionally silent during meiotic maturation. We noticed that XRN2 protein mainly localized at chromatin during meiosis, indicating that it may be involved in transcription regulation in oocytes, perhaps through ribonuclease activity–dependent RNAPII termination (45). In summary, the present study provides comprehensive data for further research of maternal mRNA degradation and identified that, XRN2, a new exoribonuclease, is responsible for partial maternal mRNA decay in mammalian oocytes.

### Ubiquitination Control of Protein Degradation in Oocytes

Over the past decade, studies targeting protein degradation in oocytes have been focused on anaphase-promoting complex or cyclosome (APC/C) and its substrates. The APC/C is involved in multiple stages of oocytes and qualifies as an essential E3 ubiquitin ligase for destruction in meiosis (60). Besides APC/C, other E3s that participate in protein degradation in oocytes are not well understood. In this study, we performed a comprehensive analysis of E3s from proteomic data. Proteins belonging to the CRLs family account for nearly half of the E3s (Fig. 6). Maturation arrest of oocytes and stabilization of proteins were found when CRLs were inhibited, indicating that CRLs are necessary in meiosis (61). As the largest and most typical complex in the CRLs family, SCF consists of SKP1, RBX1, and CUL1 as well as variable F-BOX proteins that confer substrate selectivity (62). Several F-BOX proteins, such as FBXO34 and FBXO30, have been identified as the key regulators essential for mouse oocyte maturation (5, 63). Here, we not only found that FBXO28 is indispensable for oocyte meiosis but also analyzed its potential substrates by mass spectrometry. We discovered that UBAP1 is a key protein recognized by FBXO28. FBXO28 depletion leads to an ineffective degradation of UBAP1 and, furthermore, meiotic arrest, indicating the importance of the double-layer SCF^FBXO28^-UBAP1 cascade for oocyte development. Nonetheless, the downstream targets of UBAP1 need further investigation. These findings clearly suggest that SCF^FBXO28^ is active during oocyte maturation and is crucial for proper proteins degradation.

### Limitations of Study

A strength of our study is the characterization of oocyte proteome during *in vivo* maturation and identification of the key factor essential for this process. However, there are still several limitations, as follows: (1) This study mainly used siRNA interference to investigate the function of key proteins. Owing to the limitation of knockdown efficiency, proteins cannot be completely eliminated. Thus, transgenic or gene knockout mice are necessary for performing phenotype analysis and mechanism research in the future. (2) Furthermore, the individual proteins explored in this study are mainly upregulated during maturation. Proteins with reduced expression are also critical for oocyte development and deserve further investigation.

In summary, the present study not only explored the dynamic characteristics of proteome during oocyte maturation but also provided a valuable resource for further studies to establish the molecular network controlling oocyte development.

## Data Availability Statement

All mass spectrometry data and spectral identifications have been deposited in the ProteomeXchange Consortium via the PRIDE (64) partner repository with the data set identifier PXD018777 (TMT 6-plex labeling for GV/GVBD/MII oocytes) and PXD034150 (TMTpro 12-plex labeling for oocytes after MLN4924 treatment and Fbxo28 knockdown).

## Supplemental data

This article contains supplemental data.

## Conflict of interests

The authors declare no competing interests.
