# Supplementary material for: Proteomic Profiling Reveals the Molecular Control of Oocyte Maturation
Source: Mol Cell Proteomics. 2022 Dec 7;22(1):100481. doi: 10.1016/j.mcpro.2022.100481 (PMC9823227; doi:10.1016/j.mcpro.2022.100481)
Supplement: Supplementary figure legends [file mmc1.docx]

**SUPPLEMENTAL FIGURE LEGENDS**

**Figure S1. Expression Patterns of Key Proteins in Meiosis. Related to Figure 1.**

(A) Unsupervised clustering of protein expression in three oocyte stages. (B) Relative abundance of proteins essential for the oocyte development. (C-I) Relative abundance represents proteins in each cluster. Data are expressed as the mean ± SD were analyzed for each group. For statistical analysis, a two-tailed Student’s t-test was used in all panels, compared to GV or GVBD. n.s., not significant.

**Figure S2. GSEA Analysis of DEPs. Related to Figure 2.**

(A-D) GSEA analysis of proteins related to germinal vesicle breakdown. GSEA enrichment plots of three clusters that are enriched in the GV stage (A, B & D), one cluster enriched in the GVBD stage (C). The red line means enrichment profile in GVBD, while the blue line means enrichment profile in GVBD. (E-H) GSEA was performed in GV and MII groups. GSEA enrichment plots of representative protein clusters: activation of three pathways in MII stage (E, F & G), activation of one pathway in GV stage (H). The red line means enrichment profile in MII, while the blue line means enrichment profile in GV.

**Figure S3. Relative Abundance of Cell Cycle Proteins in DEPs. Related to Figure 3.**

(A) All proteins of four complexes (centrosomal complex, centromeric complex, kinesin complex, and kinetochore complex) were identified in the proteome. (B) Relative abundance of DEPs in four complexes. (C) Quantitative RT–PCR (RT–qPCR) results showing the relative abundance of *Cep57l1* mRNA level in oocytes and somatic tissues.

(D) RT–qPCR results showing the relative abundance of *Cep57l1* and *Cep57* mRNA levels in oocytes. (E) Alignment of amino acid sequences of CEP57L1 and CEP57. The yellow line indicates similar regions of the amino acid sequence. (F) Comparison of protein structures of CEP57L1 and CEP57 predicted by I-TASSE. (G) Knockdown efficiency of *Cep57* siRNA. (H) Bright-field images of control and si*Cep57*-injected oocytes. Scale bars, 50 µm. (I) Quantitative analysis of Pb1 extrusion in control and si*Cep57*-injected oocytes. Data are expressed as the mean ± SD from three independent experiments in which at least 100 oocytes were analyzed for each group. For statistical analysis, a two-tailed Student’s t-test was used in all panels, compared to GV or control. n.s., not significant.

**Figure S4. Relative Abundance of Epigenetic Modulators in DEPs. Related to Figure 4.**

(A) Heatmap showing epigenetic modulators in DEPs. (B) Relative abundance of six proteins in histone H1. (C) Bright-field images of control and si*Sin3a*-injected oocytes. Scale bars, 50 µm. (D) Acetylation patterns of six histone sites during oocyte maturation. (E) Quantitative fluorescence analysis of histone acetylation. (F) Represent fluorescence images of histone acetylation in MI oocytes after si*Sin3a* knockdown. Data are expressed as the mean ± SD from three independent experiments in which at least 100 oocytes were analyzed for each group. For statistical analysis, a two-tailed Student’s t-test was used in all panels, compared to GV. n.s., not significant.

**Figure S5. The Function of XRN2 in Meiosis. Related to Figure 5.**

(A) Network analysis of DEPs associated with RNA metabolism. Proteins in the 5’ RNA decay pathway and 3’ RNA decay pathway are marker by blue circles. (B) Schematic diagram showing the pathways of 5’-3’ mRNA decay and 3’-5’ mRNA decay. (C) Schematic diagram showing the functions of XRN1 and XRN2 in eukaryote cells. (D) Relative abundance of *Xrn1* and *Xrn2* transcripts from transcriptome sequencing in MII oocytes. (E) Knockdown efficiency of *Xrn2* siRNA. (F) Quantitative analysis of Pb1 extrusion in control and si*Xrn2*-injected oocytes. Data are expressed as the mean ± SD from three independent experiments in which at least 100 oocytes were analyzed for each group. For statistical analysis, a two-tailed Student’s t-test was used in all panels, compared to control. n.s., not significant.

**Figure S6. Network Diagram of E1, E2, and E3. Related to Figure 6.**

(A) Schematic diagram showing the ubiquitin-proteasome pathway. (B) Network analysis of E1 ubiquitin-activating enzymes. (C) Network analysis of E2 ubiquitin-conjugating enzymes. DEPs in E2 are marker by green circles. (D) The type of E3 ubiquitin-protein ligases. (E) Network analysis of E3 ubiquitin-protein ligases (E3s). DEPs in E3s are marker by green circles.

**Figure S7. Relative Abundance of CUL1 and FBXO28 in Oocytes. Related to Figure 7.**

(A) Schematic diagram showing the cullin proteins as a scaffold of each CRLs complex.

(B) Pie plot showing the protein number of each complex in Cullin-RING ubiquitin ligases (CRLs). (C) Quantitative analysis for the transcript levels of cullin families in MII oocytes confirmed by RT-qPCR. (D) Relative abundance of cullin proteins in oocytes at different stages. (E) RT-qPCR results showing relative expression levels of *Fbxo28* in mouse somatic tissues and oocytes. (F) Transcriptome results showing relative expression levels of *Fbxo28* in mouse pre-implantation embryos. (G) Bright-field images of control and si*Fbxo28*-injected oocytes. Arrowheads point to oocytes that fail to extrude a polar body. Scale bars, 50 µm. (H) Quantitative analysis of GVBD rate in control and si*Fbxo28*-injected oocytes. (I) Quantitative analysis of Pb1 extrusion rate in control and si*Fbxo28*-injected oocytes. Data are expressed as the mean ± SD from three independent experiments in which at least 100 oocytes were analyzed for each group. For statistical analysis, a two-tailed Student’s t-test was used in all panels, compared to GV or control. * p<0.05, # p>0.05.

**Figure S8. The Function of UBAP1 in Meiosis. Related to Figure 7.**

(A) An overview of a proteomic method for identifying differential proteins in control and si*Fbxo28*-injected oocytes. (B) Venn diagram showing top 10 proteins in oocytes of MLN4924 treated group (set I) and FBXO28 knockdown group (set II). (C) Relative abundance of UBAP1 levels in si*Fbxo28*-injected oocytes and control siRNA-injected oocytes. (D) Relative abundance of UBAP1 levels in MLN4924 treated oocytes compared to DMSO treated oocytes. (E) Knockdown efficiency of *Ubap1* siRNA. (F) Quantitative analysis of Pb1 extrusion rate in control and *Ubap1* knockdown oocytes.

Data are expressed as the mean ± SD from three independent experiments in which at least 100 oocytes were analyzed for each group. For statistical analysis, a two-tailed Student’s t-test was used in all panels, compared to GV or control.
