# Supplementary figures and images for: Proteomic Profiling Reveals the Molecular Control of Oocyte Maturation
Source: Mol Cell Proteomics. 2022 Dec 7;22(1):100481. doi: 10.1016/j.mcpro.2022.100481 (PMC9823227; doi:10.1016/j.mcpro.2022.100481)

Fig S1

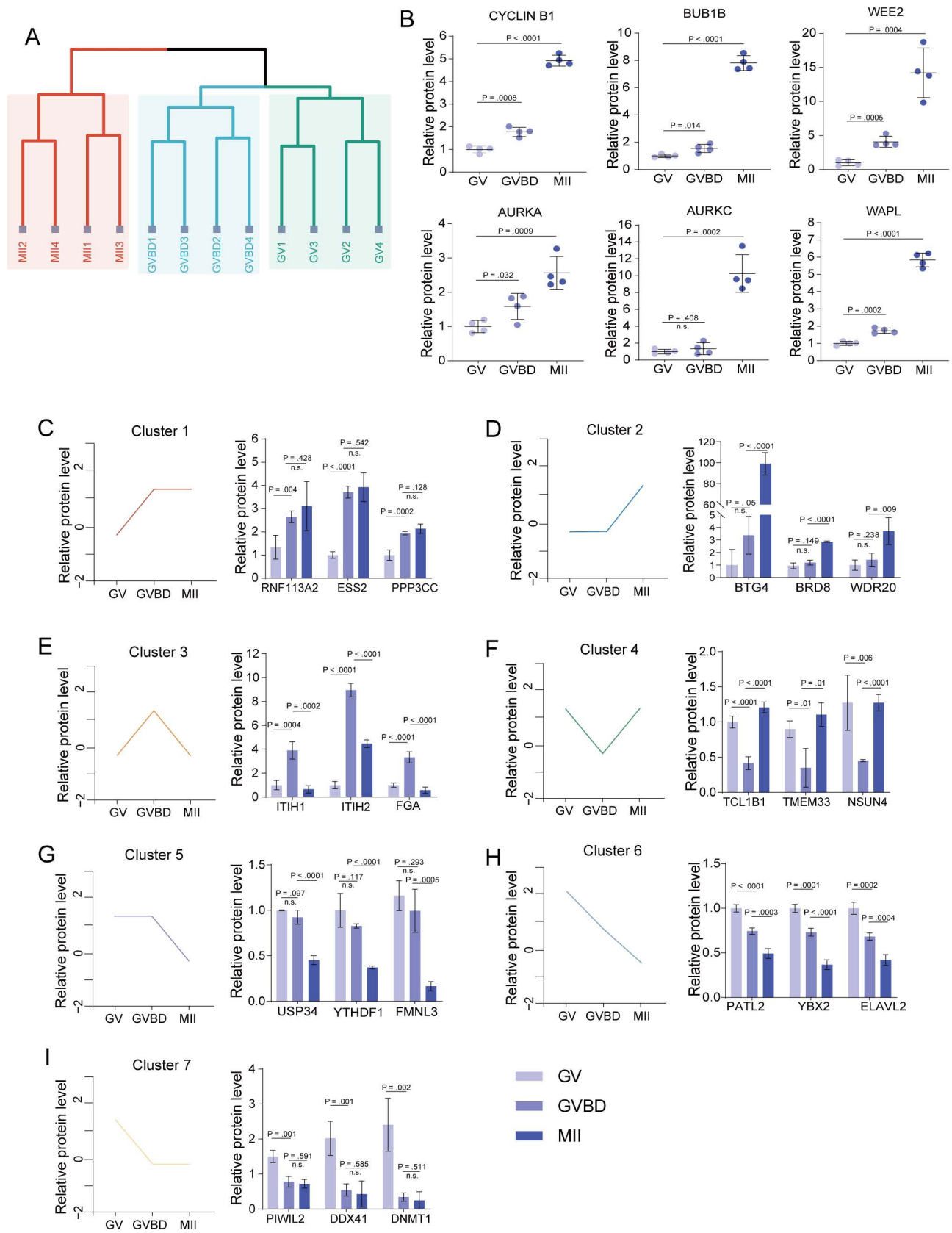

Fig S2

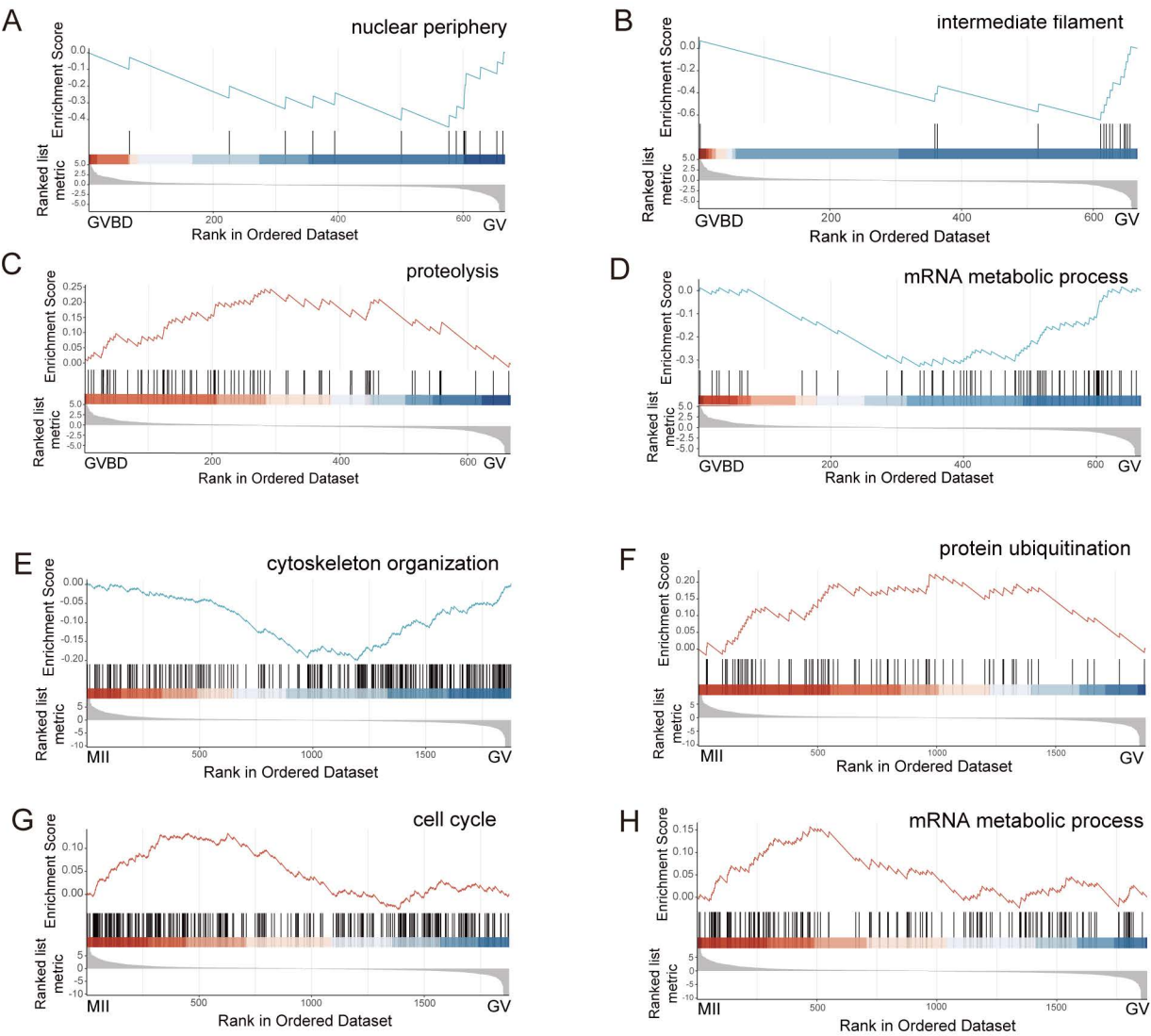

Fig S3

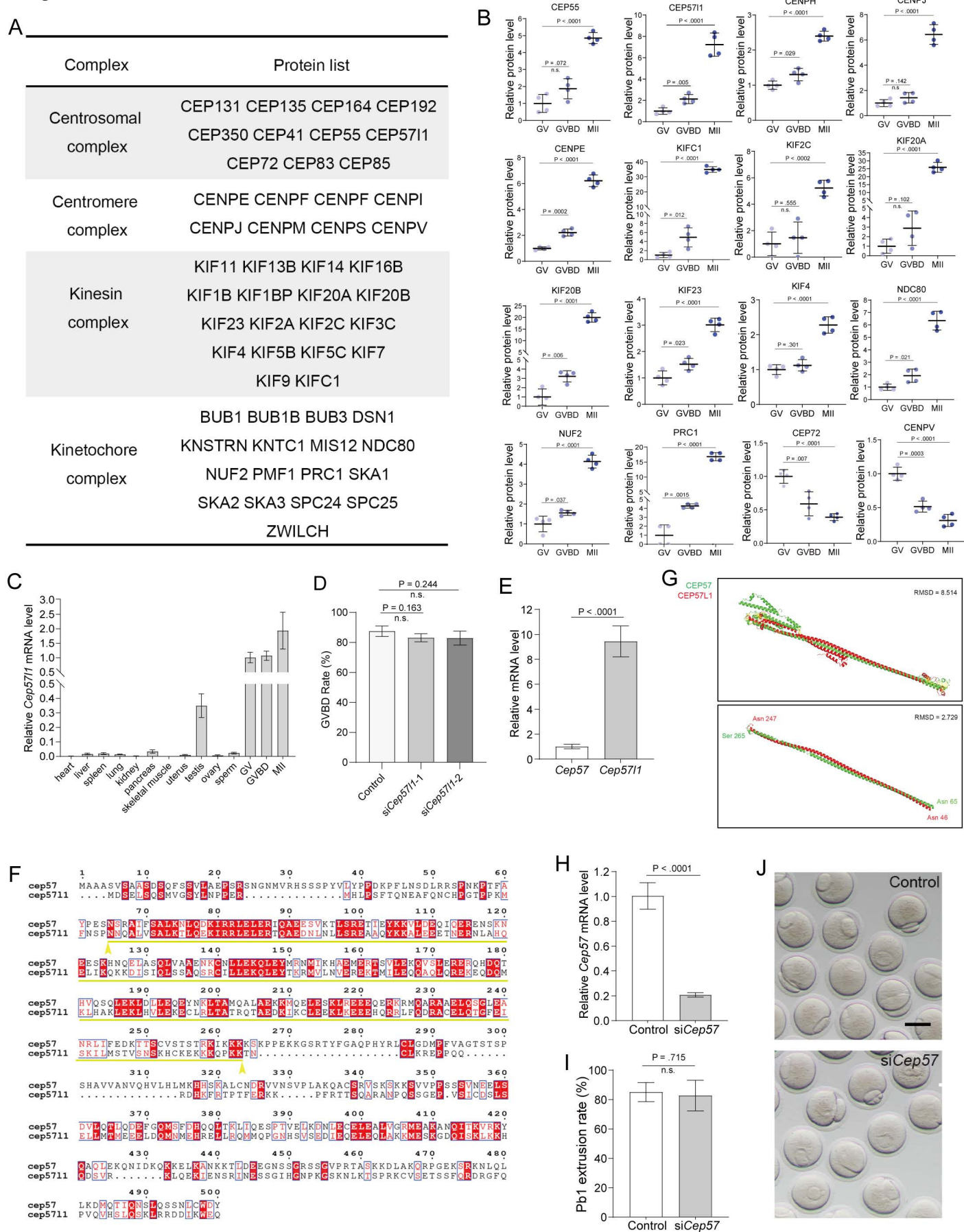

Fig S4

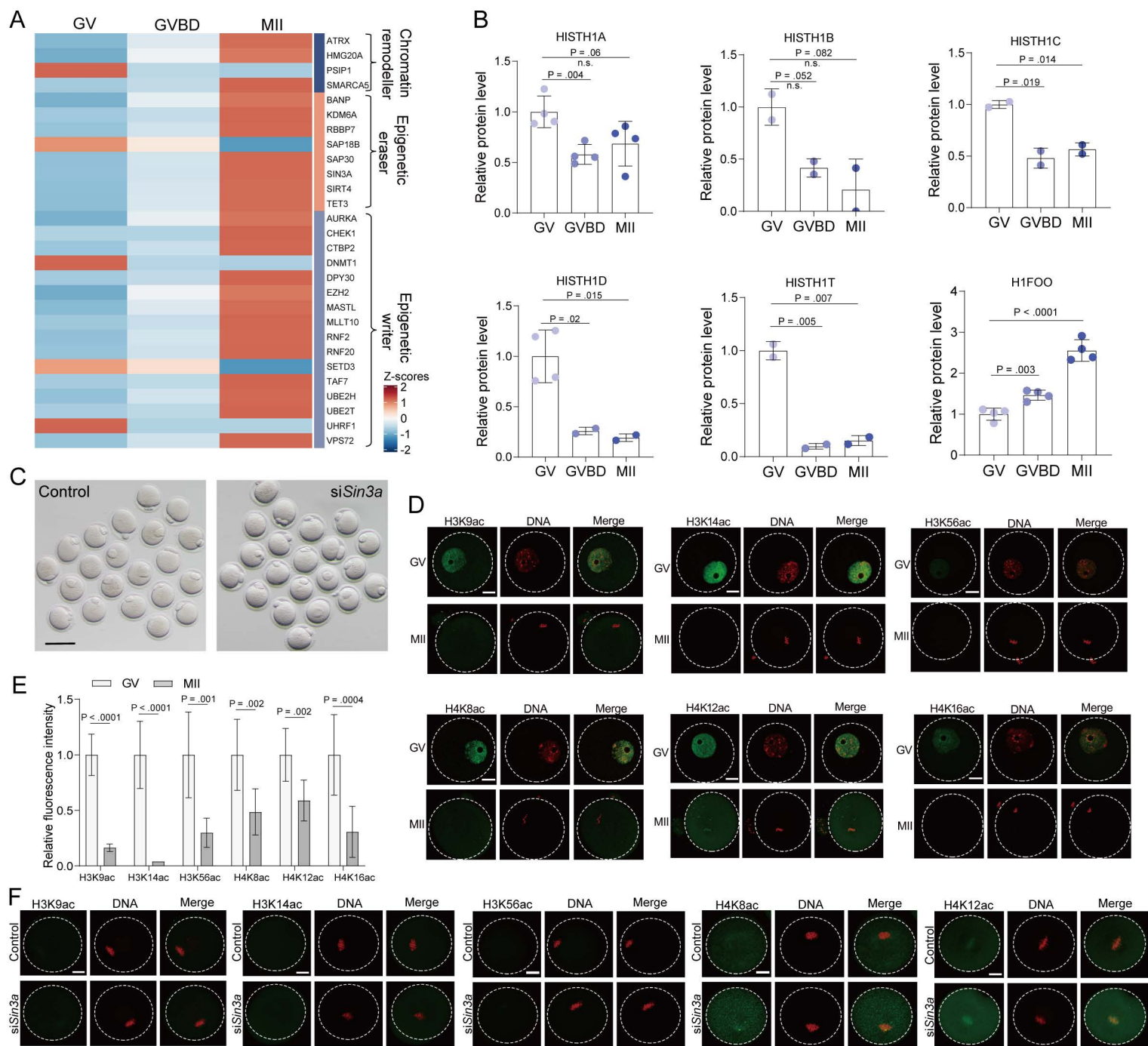

Fig S5

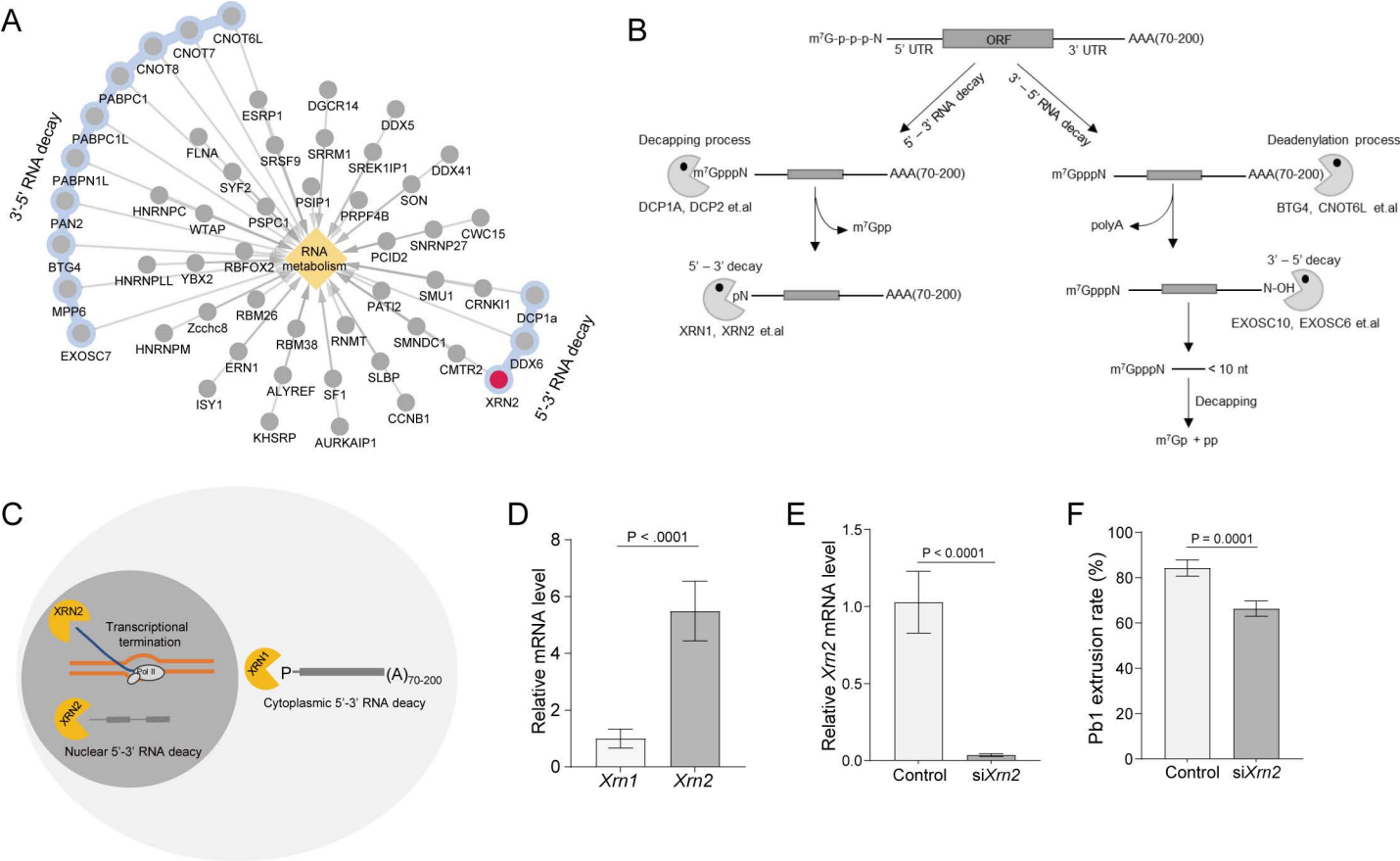

Fig S6

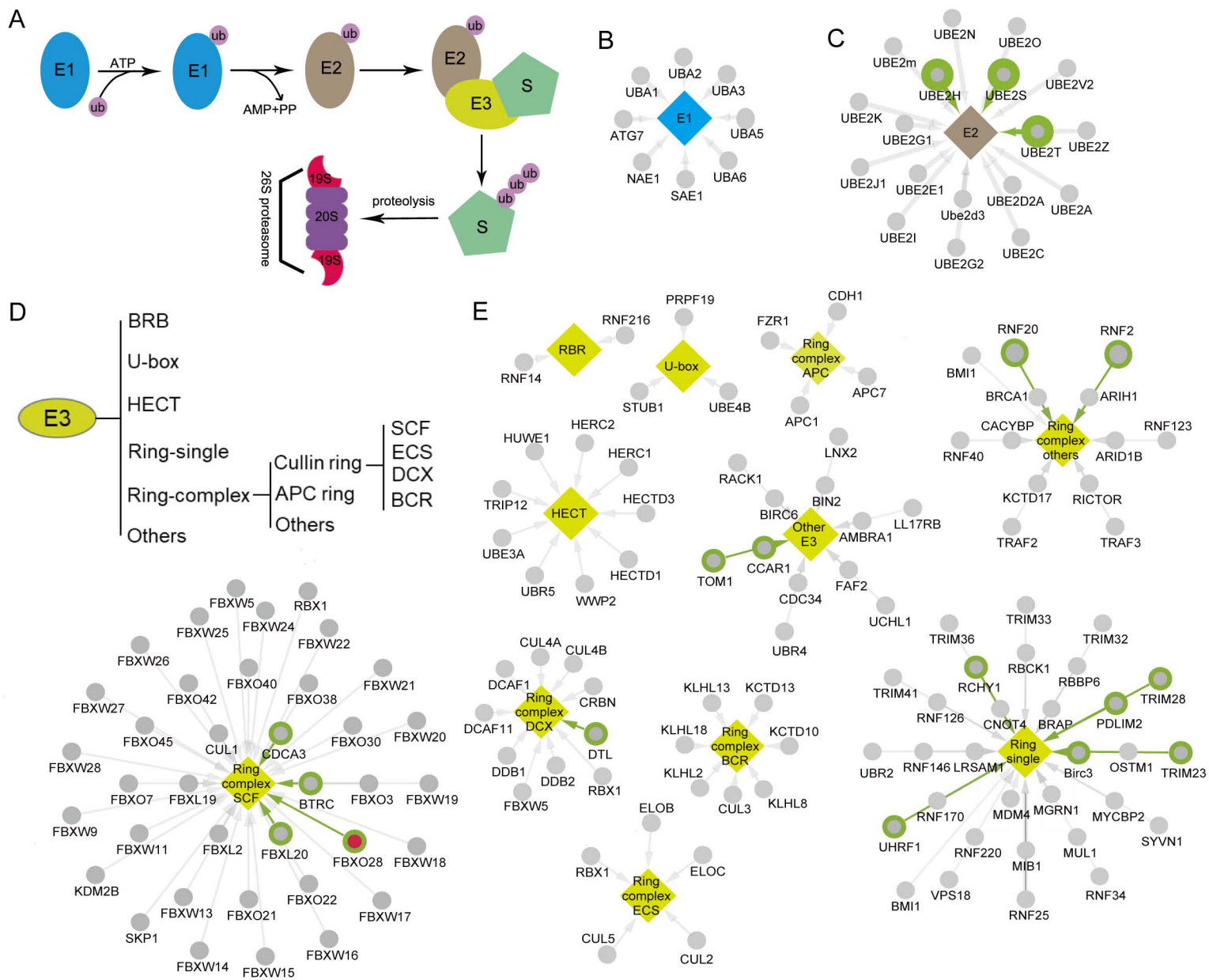

Fig S7

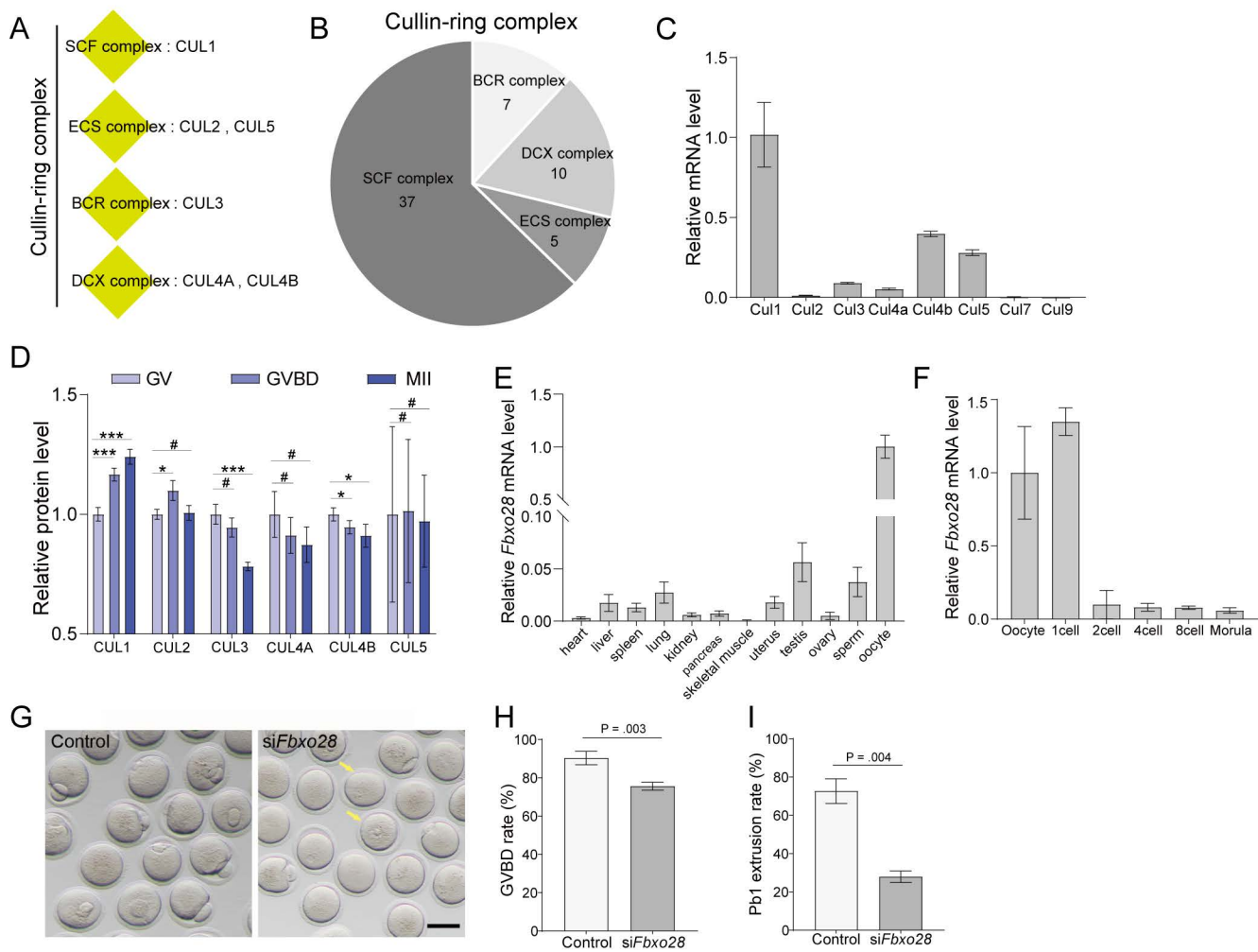

Fig S8

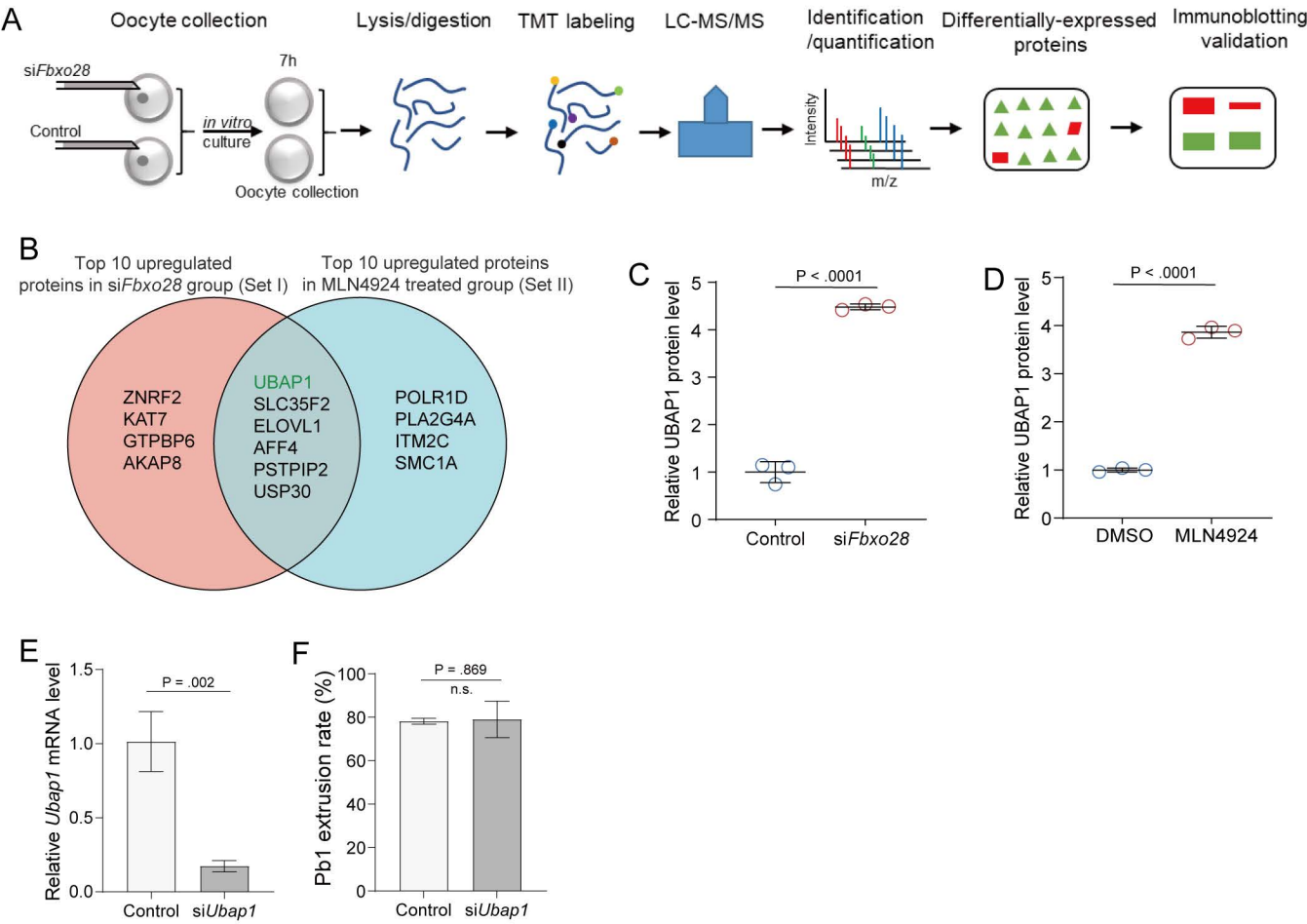

Supplement: Supplementary figures [file mmc2.pdf]
