# Supplementary material for: Proteomic Profiling Reveals the Molecular Control of Oocyte Maturation
Source: Mol Cell Proteomics. 2022 Dec 7;22(1):100481. doi: 10.1016/j.mcpro.2022.100481 (PMC9823227; doi:10.1016/j.mcpro.2022.100481)
Supplement: Table S6 [file mmc8.docx]

**ANTIBODIES**

| Antibody | Source | Cat. No |
| --- | --- | --- |
| CEP57L1 | Proteintech | 24957-1-AP |
| SIN3A | Proteintech | 14638-1-AP |
| XRN2 | Santa Cruz | sc-365258 |
| UBAP1 | Proteintech | 12385-1-AP |
| Mouse monoclonal anti-GAPDH | Proteintech | HRP-60004 |
| Mouse monoclonal anti-α-tubulin-HRP | Proteintech | HRP-66031 |
| Mouse monoclonal anti-Myc | Abcam | ab18185 |
| Goat anti-rabbit IgG-FITC | Thermo Fisher | 65-6111 |
| Goat anti-rabbit IgG-HRP | Thermo Fisher | 81-1620 |
| Goat anti-mouse IgG-HRP | Beyotime | A0216 |
| Human anti-Centromere CREST | Fitzgerald Industries International | 09C-CS1058 |
| Sheep polyclonal anti-BubR1 | Abcam | ab28193 |
| Rabbit monoclonal anti-H3K9ac | Abcam | ab32129 |
| Rabbit monoclonal anti-H3K14ac | Abcam | ab52946 |
| Rabbit polyclonal anti-H3K56ac | CST | 4243 |
| Rabbit monoclonal anti-H4K8ac | Abcam | ab45166 |
| Rabbit monoclonal anti-H4K12ac | Abcam | ab177793 |
| Rabbit monoclonal anti-H4K16ac | Abcam | ab109463 |
