# Supplementary material for: Proteomic Profiling Reveals the Molecular Control of Oocyte Maturation
Source: Mol Cell Proteomics. 2022 Dec 7;22(1):100481. doi: 10.1016/j.mcpro.2022.100481 (PMC9823227; doi:10.1016/j.mcpro.2022.100481)
Supplement: Table S7 [file mmc9.docx]

**REAGENTS**

| Reagent | Source | Cat. No |
| --- | --- | --- |
| M2 medium | Sigma-Aldrich | M7167 |
| HTF medium | Merck Millipore | MR-070 |
| KSOM medium | Merck Millipore | MR-106-D |
| Milrinone | Sigma-Aldrich | M4659 |
| MLN4924 | Selleck | S7109 |
| Mineral oil | Sigma-Aldrich | M8410 |
| Hoechst 33342 | Thermo Fisher | H3570 |
| Propidium iodide | Thermo Fisher | P3566 |
| Anti-fade medium | Vectashield | H1000 |
| Proteinase K | Thermo Fisher | EO0492 |
| AscI endonuclease | New England Biolabs | R0558S |
| FseI endonuclease | New England Biolabs | T4 DNA ligase |
| T4 DNA ligase | New England Biolabs | M0202S |
| Phusion high-fidelity DNA polymerase | New England Biolabs | M0530L |
| Arcturus PicoPure RNA Isolation Kit | Thermo Fisher | KIT0204 |
| Quantitect Reverse Transcription Kit | QIAGEN | 205311 |
| QIAquick PCR Purification Kit | QIAGEN | 28104 |
| SP6 mMESSAGE mMACHINE Kit | Thermo Fisher | AM1340 |
| Pierce ECL Western Blotting Substrate | Thermo Fisher | 32106 |
| SuperScript III First-Strand Synthesis SuperMix | Thermo Fisher | 18080-400 |
| TMT six plex Isobaric Label Reagent Set | Thermo Fisher | 90066 |
| TOP10 Competent E. coli | Tiangen | CB104 |

Note: Other chemicals and reagents not described here were all purchased from Sigma (St. Louis, MO, USA)
