# Supplementary material for: Proteomic Profiling Reveals the Molecular Control of Oocyte Maturation
Source: Mol Cell Proteomics. 2022 Dec 7;22(1):100481. doi: 10.1016/j.mcpro.2022.100481 (PMC9823227; doi:10.1016/j.mcpro.2022.100481)
Supplement: Table S8 [file mmc10.docx]

**RT-qPCR PRIMERS**

| Primer | Sequence |
| --- | --- |
| Cep57l1-F | ACTCTCCAGGAAAAGATTCGTCG |
| Cep57l1-R | GTCTCCTCCTCTAGGGCCTT |
| Cep57-F | ACGCTCCCCAAATAAGCCC |
| Cep57-R | CAGTTCCAGGCGTCGAATCT |
| Sin3a-F | GCCTCAGGTCTACAATGATTTCC |
| Sin3a-R | CCATGTCATTAGTCTGCACCTCAA |
| Xrn2-F | GCAACGATACTACAAGAACAAAT |
| Xrn2-R | CCTTCAAAGTCTGAGGCAAAT |
| Rps19-F | CAGCAGGAGTTCGTCAGAGC |
| Rps19-R | CACCCATTCGGGGACTTTCA |
| Fcf1-F | TATGCGACTATGAAGCGAATGC |
| Fcf1-R | CGCGCTCGGATCTTTCTTCT |
| Yif1a-F | TGGAGTCCACGGTTCCAAG |
| Yif1a-R | GGAGGTGCCATAGGCCATTG |
| Rpl18a-F | ACGCTTCGGGAGTACAAGGT |
| Rpl18a-R | CAGGGGTGACTTCTCAAACAC |
| Rpl6-F | GGTGAAAAAGCGCCTGATACA |
| Rpl6-R | CGGGAGTACCTGCCAATTCC |
| Rps17-F | CCAAGACCGTGAAGAAGGCTG |
| Rps17-R | GCTGGGGATAATGGCGATCT |
| Ppil3-F | CTGTGAGAGAACACCCAAAACA |
| Ppil3-R | TTTGAACCATGAAGCCCTTGAT |
| Rpl10-F | CGTGGTGTCCCTGATGCTAAG |
| Rpl10-R | GTTGGCACAAATACGGGCAG |
| Rpl10a-F | ATGAGCAGCAAAGTCTCACG |
| Rpl10a-R | GGTCGTAGTTCTTCAGGCTGAT |
| Rpl19-F | ATGAGTATGCTCAGGCTACAGA |
| Rpl19-R | GCATTGGCGATTTCATTGGTC |
| Snrpd2-F | AAACCCAAGAGTGAGATGACCC |
| Snrpd2-R | ATGAGCACTTGCGTGTTGTTC |
| Cul1-F | CTCAGTTTGTTGGCTTGGAGT |
| Cul1-R | TGGAGAATCGGTAATCTTCCCA |
| Cul2-F | GATGGTAGCAGACCACTTACAG |
| Cul2-R | GACACAGCACGGAGTAAGAC |
| Cul3-F | CACATCGAAACTGGAAGGAATG |
| Cul3-R | TGTGAGATCAACACCACCTAAA |
| Cul4a-F | TCTGGATCGAACCTACGTCCT |
| Cul4a-R | CACCATCCTGTCGCTGATGAT |
| Cul4b-F | TGCTGGCAAAACCACTGTAGG |
| Cul4b-R | CCAAATGGAGGGTAGCATTGAA |
| Cul5-F | TGCTGAGAGATTAGGGGAAGC |
| Cul5-R | TGCTGTAAATATGAGGGTGCCT |
| Cul7-F | GACCCAGATCCTTCTGTCATTG |
| Cul7-R | GTGTTCCGTGAGAGTAGCCTG |
| Cul9-F | CGTTGAGAGAGAAGCTCGTGA |
| Cul9-R | ACGCCATTCATGCTTGGTCA |
| Fbxo28-F | ATGCAGCTCTTCTCCAAGCA |
| Fbxo28-R | TTCGCCTATGATCTGGGTCT |
| Ubap1-F | AAGAAGTTGGGGACAGATGTTC |
| Ubap1-R | CAATAGGCAGACCCACTTTCG |
| GAPDH-F | CTTTGTCAAGCTCATTTCCTGG |
| GAPDH-R | TCTTGCTCAGTGTCCTTGC |

**PCR PRIMERS**

| Primer | Sequence |
| --- | --- |
| Ubap1-F | CAGGCCGGCCAGCGGCATTCAGGTTCTAAATG |
| Ubap1-R | GAGGCGCGCCGGCCAGGTCTCAGCTGGCT |

Note: Yellow marker is the endonuclease sites
