# Supplementary material for: Proteomic Profiling Reveals the Molecular Control of Oocyte Maturation
Source: Mol Cell Proteomics. 2022 Dec 7;22(1):100481. doi: 10.1016/j.mcpro.2022.100481 (PMC9823227; doi:10.1016/j.mcpro.2022.100481)
Supplement: Table S9 [file mmc11.docx]

**siRNAs**

| siRNA | Sequence |
| --- | --- |
| Cep57l1-413 | 5’-GCAGTATAAGAAGGCCCTAdTdT-3’ |
| Cep57l1-671 | 5’-GCTCCATGTCCTAGAGAAAdTdT-3’ |
| Cep57-262 | 5’-GCTGAACCATCAAGGTCTAdTdT-3 |
| Xrn2-1763 | 5’-GCUGGGUUCUUCGCUAUUAdTdT-3’ |
| Sin3a-893 | 5’-CCGAGUGUCCCAGCUAUUUdTdT-3’ |
| Fbxo28-371 | 5’- GCAGCAGAAACAGCUUCAAdTdT-3 |
| Ubap1-433 | 5’- GUGGGCUGAAGAUAUUAAAdTdT-3’ |
